# Defects in TRPM7 channel function deregulate thrombopoiesis through altered cellular Mg^2+^ homeostasis and cytoskeletal architecture

**DOI:** 10.1038/ncomms11097

**Published:** 2016-03-29

**Authors:** Simon Stritt, Paquita Nurden, Remi Favier, Marie Favier, Silvia Ferioli, Sanjeev K. Gotru, Judith M M. van Eeuwijk, Harald Schulze, Alan T. Nurden, Michele P. Lambert, Ernest Turro, Stephanie Burger-Stritt, Masayuki Matsushita, Lorenz Mittermeier, Paola Ballerini, Susanna Zierler, Michael A. Laffan, Vladimir Chubanov, Thomas Gudermann, Bernhard Nieswandt, Attila Braun

**Affiliations:** 1 Chair of Experimental Biomedicine, University Hospital, University of Würzburg, Josef-Schneider-Strasse 2, 97078 Würzburg, Germany; 2 Rudolf Virchow Centre, University of Würzburg, Josef-Schneider-Strasse 2, 97078 Würzburg, Germany; 3 Institut Hospitalo-Universitaire LIRYC, Plateforme Technologique d'Innovation Biomédicale, Hôpital Xavier Arnozan, Avenue du Haut Lévêque, 33604 Pessac, France; 4 Assistance Publique—Hôpitaux de Paris, Haematological Laboratory, Armand Trousseau Children Hospital, 26 Avenue du Docteur Arnold-Netter, 75012 Paris, France; 5 Inserm U1170, Gustave Roussy, University Paris Sud, 114 Rue Edouard Vaillant, 94805 Villejuif, France; 6 Inra, UMR_INRA 1260, 27 Boulevard Jean Moulin, 13385 Marseille, France; 7 Aix Marseille Université, 58 Boulevard Charles Livon, 13284 Marseille, France; 8 Inserm UMR_S 1062, 27 Boulevard Jean Moulin, 13385 Marseille, France; 9 Walther-Straub-Institute for Pharmacology and Toxicology, Ludwig-Maximilians University Munich, Goethestraße 33, 80336 Munich, Germany; 10 Division of Haematology, Children's Hospital of Philadelphia, 3401 Civic Center Blvd, Philadelphia, Pennsylvania 19104, USA; 11 Department of Paediatrics, Perelman School of Medicine at the University of Pennsylvania, 3400 Civic Center Blvd, Philadelphia, Pennsylvania 19104, USA; 12 Department of Haematology, University of Cambridge, Cambridge Biomedical Campus, Francis Crick Ave, Cambridge CB2 0S, UK; 13 NHS Blood and Transplant, Cambridge Biomedical Campus, Francis Crick Ave, Cambridge CB2 0S, UK; 14 Medical Research Council Biostatistics Unit, Cambridge Institute of Public Health, Cambridge Biomedical Campus, Robinson Way, Cambridge CB2 0SR, UK; 15 NIHR BioResource—Rare Diseases, Cambridge University Hospitals, Cambridge Biomedical Campus, Hills Rd, Cambridge CB2 0QQ, UK; 16 Endocrinology and Diabetes Unit, Department of Internal Medicine I, University Hospital of Würzburg, Oberdürrbacher Strasse 6, 97080 Würzburg, Germany; 17 Department of Molecular & Cellular Physiology, Graduate School of Medicine, University of the Ryukyus, 207 Uehara, Okinawa 903-0215, Japan; 18 Centre for Haematology, Hammersmith Campus, Imperial College Academic Health Sciences Centre, Imperial College London, Du Cane Road, London SW7 2AZ, UK; 19 Imperial College Healthcare NHS Trust, Du Cane Road, London SW7 2AZ, UK; 20 Comprehensive Pneumology Center Munich (CPC-M), German Center for Lung Research, Max-Lebsche-Platz 31, 81377 Munich, Germany; 21 DZHK (German Centre for Cardiovascular Research), Munich Heart Alliance, Lazarettstraße 36, 80636 Munich, Germany

## Abstract

Mg^2+^ plays a vital role in platelet function, but despite implications for life-threatening conditions such as stroke or myocardial infarction, the mechanisms controlling [Mg^2+^]_i_ in megakaryocytes (MKs) and platelets are largely unknown. Transient receptor potential melastatin-like 7 channel (TRPM7) is a ubiquitous, constitutively active cation channel with a cytosolic α-kinase domain that is critical for embryonic development and cell survival. Here we report that impaired channel function of TRPM7 in MKs causes macrothrombocytopenia in mice (*Trpm7*^*fl/fl-Pf4Cre*^) and likely in several members of a human pedigree that, in addition, suffer from atrial fibrillation. The defect in platelet biogenesis is mainly caused by cytoskeletal alterations resulting in impaired proplatelet formation by *Trpm7*^*fl/fl-Pf4Cre*^ MKs, which is rescued by Mg^2+^ supplementation or chemical inhibition of non-muscle myosin IIA heavy chain activity. Collectively, our findings reveal that TRPM7 dysfunction may cause macrothrombocytopenia in humans and mice.

## Abstract (web summary)

Although Mg^2+^ is vital for platelet activation and aggregation, its regulation in these cells is still largely unknown. Here, the authors show that TRPM7, a cation channel and a protein kinase, regulates thrombopoiesis and platelet size by affecting the cytoskeleton of these cells in mice and humans.

## Graphical abstract

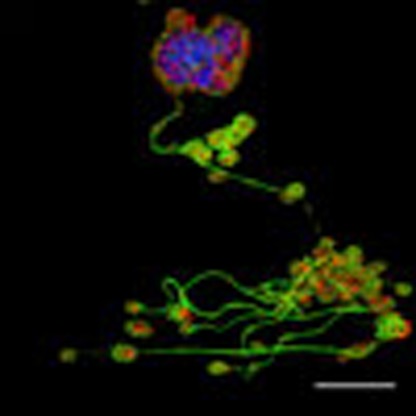

Platelets are continuously produced from megakaryocytes (MKs) in the bone marrow by a cytoskeleton-driven process of which the molecular regulation is not fully understood. MKs extend long cytoplasmic protrusions into bone marrow sinusoids, where larger fragments, so-called preplatelets, are shed and further divide within the circulation to give rise to platelets (Supplementary Movie 1)^1^,^2^,^3^.

Transient receptor potential melastatin-like 7 (TRPM7) channel and kinase domain, but not its kinase activity, are critical for embryonic development^4^,^5^,^6^ and knockdown or cell-specific TRPM7 knockout approaches give rise to impaired cytoskeletal organization, cell migration, proliferation, polarization and survival. These defects could partially be explained by increased non-muscle myosin IIA heavy chain (NMMIIA)-mediated contractility of the actin cytoskeleton^5^,^7^,^8^,^9^,^10^,^11^,^12^,^13^. Of note, among other substrates, the kinase domain of TRPM7 phosphorylates annexin I and NMMIIA, thus interfering with cell survival and cytoskeletal rearrangements^14^,^15^. Interestingly, several variants of NMMIIA similarly altered the contractility of the actomyosin complex in MKs, thereby interfering with proplatelet formation in humans and mice^16^. During megakaryopoiesis, NMMIIA activity is suppressed by phosphorylation of its C-terminus, enabling MK polyploidisation and ultimately proplatelet formation^17^. However, for proper platelet fission and sizing, NMMIIA needs to be re-activated under shear stress in the circulation^16^,^18^. Although both kinase and channel activity of TRPM7 have been proposed to regulate cytoskeletal dynamics, channel activity alone was sufficient to restore cell polarization, morphology and migration^10^,^13^,^19^, suggesting a critical role of cations therein. Consequently, the differential role of TRPM7 channel versus kinase activity in the regulation of the cytoskeleton still remains unclear. Moreover, TRPM7 has been implicated as a key regulator of signal conductance in the murine heart by regulating the expression of different pacemaker channels, such as HCN4 (ref. 20). Although TRPM7-mediated cation influx has been detected in MKs (ref. 21), its role in thrombopoiesis has not been investigated to date.

Here we report that impaired channel function but not kinase activity of TRPM7 in MKs causes macrothrombocytopenia in *Trpm7*^*fl/fl-Pf4Cre*^ mice and likely in several members of a human pedigree, which, in addition, feature atrial fibrillation. The impaired proplatelet formation is associated with cytoskeletal alterations due to increased actomyosin contractility and can be rescued by either Mg^2+^ supplementation or chemical inhibition of NMMIIA activity. Collectively, our findings reveal TRPM7 dysfunction as a novel cause of macrothrombocytopenia in mice and potentially in humans too.

## Results

### Defects in TRPM7 cause macrothrombocytopenia

**Figure 1:**
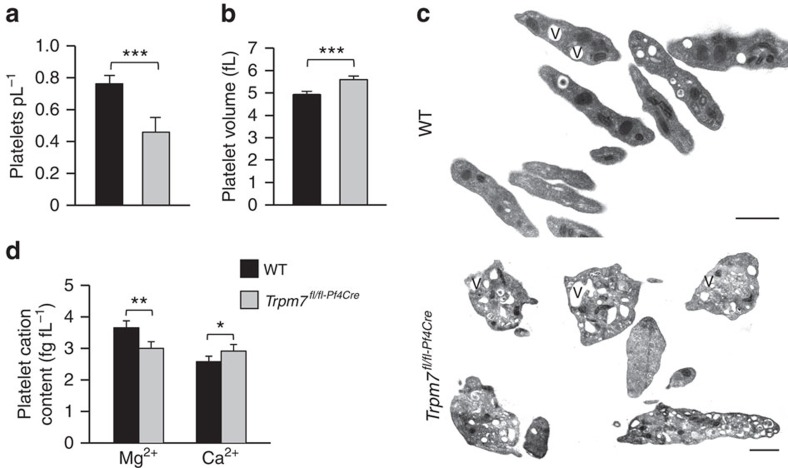
TRPM7 deficiency causes mild macrothrombocytopenia in mice. Moderately reduced peripheral platelet counts (**a**) and significantly increased platelet volume (**b**) were quantified with an automated cell analyser. Values are mean±s.d. (*n*=7). (**c**) Transmission electron microscopy (TEM) analysis shows discoid WT platelets, but enlarged and round resting platelets of *Trpm7*^*fl/fl-Pf4Cre*^ mice. V, vacuole. Scale bar, 1 μm. (**d**) Total platelet cation content was determined by inductively coupled plasma mass spectrometry. Values are mean±s.d. (*n*=5). Unpaired Student's *t*-test; ***P<0.001; **P<0.01; *P<0.05.

We identified TRPM7 as the key Mg^2+^ channel and magnesium transporter 1 (MagT1) to be expressed in murine platelets (Supplementary Fig. 1a) and generated MK- and platelet-specific TRPM7 knockout mice (Supplementary Fig. 1b,c). The absence of TRPM7 currents in patch clamp measurements confirmed the efficacy of the targeting strategy in primary bone marrow MKs (Supplementary Fig. 1d). Unexpectedly, these mice displayed a macrothrombocytopenia (Fig. 1a,b) with enlarged and spherical platelets, often containing large vacuoles as revealed by electron microscopy (Fig. 1c). In contrast, mice carrying a kinase-dead K1646R mutation in *Trpm7* (ref. 6; *Trpm7*^*KI*^) showed normal platelet counts, size and morphology, thus suggesting that the lack of TRPM7 channel function rather than its kinase activity accounts for the macrothrombocytopenia in the mutant mice (Supplementary Fig. 2a–e). In line with this notion, intracellular Mg^2+^ concentrations in *Trpm7*^*fl/fl-Pf4Cre*^ platelets, but not in *Trpm7*^*KI*^ platelets, were decreased (Fig. 1d; Supplementary Fig. 2f)^6^.

### Impaired proplatelet formation causes thrombocytopenia

**Figure 2:**
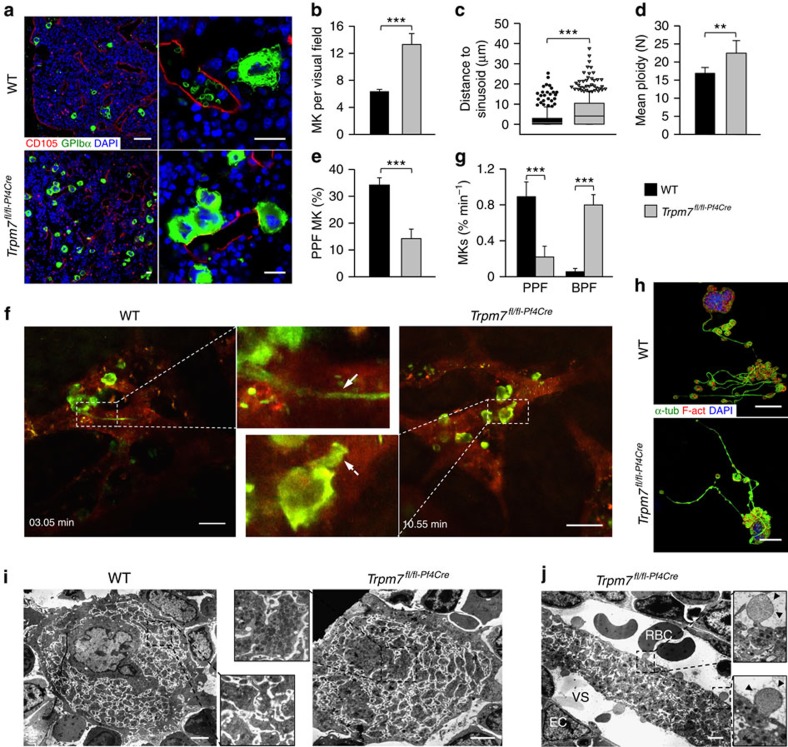
Cytoskeletal alterations account for the macrothrombocytopenia. (**a**) Confocal microscopy images of immunostained bone marrow sections. Scale bars, 50 μm (left panel. Scale bars, 15 μm (right panel). MKs, proplatelets and platelets are identified by GPIb staining (green). Endoglin staining (red) labels vessels; 4′,6-diamidino-2-phenylindole (DAPI) stains nuclei (blue). (**b**) Quantification of bone marrow MKs per visual field (328 × 246 μm). Values are mean±s.d. (*n*=6; each 20 visual fields were analysed). (**c**) MK localization was quantified as their distance from bone marrow sinusoids. Values are mean±s.d. (*n*=6; 300 MKs). Wilcoxon–Mann–Whitney test; ***P<0.001. (**d**) Mean ploidy of primary bone marrow MKs. Values are mean±s.d. (*n*=6). (**e**) Percentage of proplatelet-forming foetal liver-derived MKs *in vitro* on day 4 of culture. Values are mean±s.d. (*n*=7). (**f**) Intravital two-photon microscopy of bone marrow MKs in the skulls of WT and *Trpm7*^*fl/fl-Pf4Cre*^ mice. Arrow shows a normal sized proplatelet in a WT bone marrow sinusoid (PPF); dashed arrow indicates bulky proplatelet in the sinus of a mutant mouse (BPF). Rhodamine dextran labels vessels (red) and anti-GPIX antibodies label MKs and platelets (green). Scale bars, 25 μm. (**g**) Quantification of proplatelet formation *in vivo* by intravital two-photon microscopy. Proplatelet formation was quantified during a period of 13 h (27 movies each 30 min duration) in WT and 6.5 h (13 movies each 30 min duration) in *Trpm7*^*fl/fl-Pf4Cre*^ mice and normalized to the cell number per visual observed field. Values are mean±s.d. (*n*=12 versus 6). (**h**) Confocal microscopy of *in vitro* cultured foetal liver-derived MKs revealed an increased content of microtubules in rarely found and less branched proplatelets of *Trpm7*^*fl/fl-Pf4Cre*^ MKs. α-tubulin (green) and F-actin (red) stain the cytoskeleton. DAPI, blue. Scale bar, 25 μm. (**i**,**j**) Transmission electron microscopy (TEM) analysis of bone marrow MKs. *Trpm7*^*fl/fl-Pf4Cre*^ MKs display an irregular distribution of granules, tortuous membranes (**i**) and proplatelet structure (**j**). EC, endothelial cell; VS, vascular sinusoid; RBC, red blood cell. Scale bars, 2.5 μm. All images are representative of at least five animals. Unpaired Student's *t*-test (if not stated otherwise); ***P<0.001; **P<0.01.

A mildly reduced platelet lifespan in *Trpm7*^*fl/fl-Pf4Cre*^ mice (*t*½=43.6 h for wild type (WT) versus *t*½=35.7 h for *Trpm7*^*fl/fl-Pf4Cre*^ mice), however, was insufficient to explain the reduced platelet count and was not associated with altered platelet terminal galactose levels (Supplementary Fig. 3a,b). Immunostaining of whole-femora bone marrow sections (Fig. 2a) revealed an increased number of MKs in the mutant mice (6.3±0.3 for WT versus 13.3±1.6 for *Trpm7*^*fl/fl-Pf4Cre*^ mice; Fig. 2b). The MKs in *Trpm7*^*fl/fl-Pf4Cre*^ mice were also located further from bone marrow sinusoids than in controls (Fig. 2c) suggesting impaired migration of MK-precursors to bone marrow sinusoids. Although splenomegaly was not observed in *Trpm7*^*fl/fl-Pf4Cre*^ mice, we found an increased number of MKs in an expanded red pulp in the spleen and in line with the increased MK numbers, plasma thrombopoietin levels were decreased, thus further indicating deregulated megakaryopoiesis (Supplementary Fig. 4a–d).

In contrast to a previous report on a neuroblastoma cell line^22^, the formation of podosomes, F-actin rich structures that are thought to serve cell migration and proplatelet protrusion^23^, was unaltered in *Trpm7*^*fl/fl-Pf4Cre*^ MKs (Supplementary Fig. 5a) suggesting that other defects must account for their more distant localization from bone marrow sinusoids (Fig. 2c). Interestingly, mutant MKs displayed an increased mean ploidy as compared with controls (16.9 N±1.6 N in controls versus 22.4 N±3.4 N for *Trpm7*^*fl/fl-Pf4Cre*^ mice; Fig. 2d) thus excluding impaired MK maturation as the cause of thrombocytopenia. Despite the increased ploidy *in vivo*, we found a decreased proplatelet formation for both foetal liver- (Fig. 2e) and bone marrow-derived (Supplementary Fig. 5b) *Trpm7*^*fl/fl-Pf4Cre*^ MKs *in vitro*. This was further confirmed *in vivo* by intravital two-photon microscopy of the bone marrow (0.88% min^−1^±0.16% min^−1^ of WT MKs formed proplatelets versus 0.23% min^−1^±0.12% min^−1^ of *Trpm7*^*fl/fl-Pf4Cre*^ MKs), which revealed that *Trpm7*^*fl/fl-Pf4Cre*^ MKs preferentially formed short and bulky proplatelet protrusions (0.85% min^−1^±0.13% min^−1^ of observed MKs in *Trpm7*^*fl/fl-Pf4Cre*^ mice versus 0.05% min^−1^±0.04% min^−1^ in WT mice) that remained attached to the cell body during the observation period (Fig. 2f,g; Supplementary Movies 1, 2, 3).

The actin and microtubule cytoskeleton are critical for proper proplatelet formation^2^. We therefore analysed the cytoskeletal architecture of *Trpm7*^*fl/fl-Pf4Cre*^ MKs and found an increased content and aberrant organization of microtubules in proplatelet-forming, resting and spread MKs (Fig. 2h; Supplementary Fig. 6a–c). In support of this, extraction of the microtubule cytoskeleton of resting bone marrow-derived MKs by ultracentrifugation revealed an increased microtubule content in the Triton X100 insoluble pellet fraction (Supplementary Fig. 6d,e). Of note, increased microtubule stability is associated with post-translational modifications of α-tubulin that include detyrosination (Glu-tub) and acetylation (ac-tub) which could account for the increased number of microtubules in *Trpm7*^*fl/fl-Pf4Cre*^ MKs (ref. 24). Nonetheless, we found an increased prevalence of highly dynamic tyrosinated (Tyr) microtubules as evidenced by lower Glu-/Tyr- (2.5±0.3 for control versus 1.5±0.2 for *Trpm7*^*fl/fl-Pf4Cre*^ MKs) and ac-/Tyr-tubulin ratios (2.2±0.2 for control versus 1.6±0.2 for samples of *Trpm7*^*fl/fl-Pf4Cre*^ mice), suggesting that accelerated microtubule assembly/dynamics accounted for the observed alterations (Supplementary Fig. 6d–g). Moreover, in line with the critical role of microtubules in trafficking of intracellular cargo, electron microscopy revealed a non-homogeneous distribution of granules, tortuous membrane complexes and aberrantly sized proplatelets in mutant MKs (Fig. 2i; Supplementary Fig. 7). Furthermore, we found thick and densely packed proplatelets in bone marrow sinusoids with signs of apoptosis reflecting impaired proplatelet fragmentation and release of preplatelets from *Trpm7*^*fl/fl-Pf4Cre*^ MKs (Fig. 2f,j; Supplementary Movies 2 and 3). In line with the impaired proplatelet formation (Fig. 2e,g; Supplementary Fig. 5b and Supplementary Movies 2 and 3), the recovery of the platelet count after antibody-induced platelet depletion was slightly delayed in *Trpm7*^*fl/fl-Pf4Cre*^ mice (Supplementary Fig. 8). The pronounced increase above initial platelet counts might be attributed to the increased number of MKs in *Trpm7*^*fl/fl-Pf4Cre*^ mice and alternative platelet release mechanisms as recently shown by Nishimura *et al*.^25^.

### Altered NMMIIA activity impairs proplatelet formation

**Figure 3:**
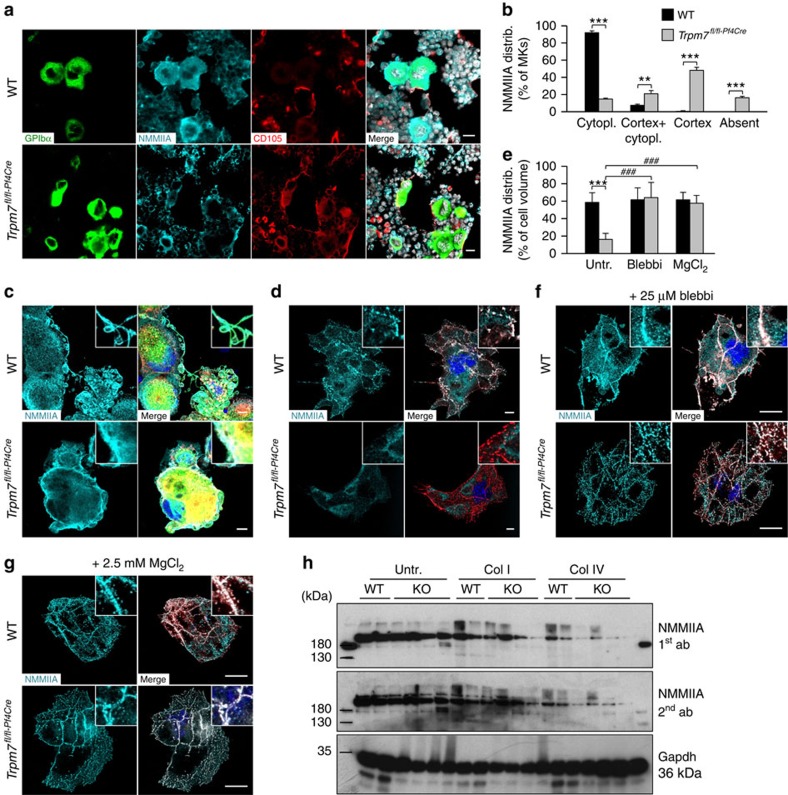
Altered localization and accelerated degradation of NMMIIA in MKs. (**a**) Confocal microscopy images of immunostained bone marrow sections. Scale bars, 10 μm. MKs, proplatelets and platelets are identified by GPIb staining (green). NMMIIA is highlighted in cyan. Endoglin staining (red) labels vessels; 4′,6-diamidino-2-phenylindole (DAPI) stains nuclei (grey). (**b**) Quantification of NMMIIA distribution in primary bone marrow MKs *in situ*. Cytopl, homogeneous cytoplasmic distribution; cortex+cyotpl, homogeneous cytoplasmic distribution and some accumulation of NMMIIA at the cell cortex; cortex, accumulation of NMMIIA at the cell cortex; absent, no NMMIIA staining was detected. Values are mean±s.d. (*n*=5; 150 MKs). (**c**–**g**) Localization of NMMIIA (cyan) in proplatelet-forming foetal liver-derived MKs on day 4 of culture (**c**) and on collagen I (50 μg ml^−1^) spread (3 h) bone marrow-derived MKs (**d**–**g**). The MK cytoskeleton was stained for α-tubulin (green) and F-actin (red). DAPI, blue. (**e**) Quantification of the relative NMMIIA content on confocal microscopy images of spread MKs. Values are mean±s.d. (*n*=5; 50 MKs per condition). Pretreatment of bone marrow-derived MKs with 25 μM blebbistatin (**e**,**f**) or 2.5 mM MgCl_2_ (**e**,**g**) prevented the degradation of NMMIIA (**b**) and restored its localization to podosomes. Scale bars, 10 μm (**a**,**b**). Scale bars, 25 μm (**c**,**d**). (**h**) Bone marrow-derived MKs of WT and *Trpm7*^*fl/fl-Pf4Cre*^ mice were cultured for 48 h on a collagen I- or IV-coated (each 10 μg cm^−2^) surfaces, lysed and NMMIIA prevalence was analysed by immunoblotting using two different antibodies (1st and 2nd ab). All images are representative of at least five animals. Unpaired Student's *t*-test; ***^,###^*P*<0.001; **P<0.01.

NMMIIA has been described as a downstream effector of TRPM7 kinase^14^,^22^ and importantly, abnormal function of NMMIIA has been associated with impaired formation and fragmentation of proplatelets in humans and mice^16^. Moreover, Mg^2+^ was recently shown to modify NMMIIA activity by regulating ADP release and its affinity to actin filaments, thus further linking TPRM7 channel to NMMIIA function^26^. Strikingly, analysis of NMMIIA localization in MKs on whole-bone-marrow sections *in situ* revealed a homogeneous distribution in the cell body of control (92.1±2.0% in WT versus 14.7±1.1% in *Trpm7*^*fl/fl-Pf4Cre*^ mice), while it predominated in the cell cortex of *Trpm7*^*fl/fl-Pf4Cre*^ MKs (0.4±0.8% in WT versus 48.1±3.6% in *Trpm7*^*fl/fl-Pf4Cre*^ mice; Fig. 3a,b). A similar distribution pattern was found *in vitro* for foetal liver-derived MKs where in addition a significant accumulation of NMMIIA in proplatelets of mature WT MKs was observed (Fig. 3c). Moreover, NMMIIA localization was also altered in *Trpm7*^*fl/fl-Pf4Cre*^ platelets. However, in contrast to MKs, NMMIIA predominated in the cell cortex of WT platelets reminiscent of the marginal band, whereas it was homogeneously distributed in platelets of *Trpm7*^*fl/fl-Pf4Cre*^ mice (Supplementary Fig. 9a–c). This observation is in line with a report on resting foreskin fibroblasts in which NMMIIA and α-tubulin staining overlapped extensively at the cell cortex under resting, non-contractile conditions. However, on induction of cell migration and activation of NMMIIA, the overlap of NMMIIA and α-tubulin staining decreased, allowing NMMIIA to exert its contractile effects on the actin cytoskeleton^27^. To further analyse the cause of the absent NMMIIA staining and the altered localization to the cell cortex in *Trpm7*^*fl/fl-Pf4Cre*^ MKs *in situ*, we next allowed bone marrow-derived MKs to spread on a collagen type I-coated surface. Surprisingly, on spreading of *Trpm7*^*fl/fl-Pf4Cre*^ MKs (Fig. 3d,e) or platelets (Supplementary Fig. 10a–d) we found a rapid degradation of NMMIIA that could be rescued by pretreatment with the NMMIIA inhibitor blebbistatin or by Mg^2+^ supplementation (Fig. 3e–g; Supplementary Fig. 10e–h). Based on these findings we speculated that deregulated [Mg^2+^]_i_ in *Trpm7*^*fl/fl-Pf4Cre*^ cells may cause an increased activity of NMMIIA resulting in its rapid degradation on cell stimulation. In agreement with the increased co-localization of NMMIIA to actin filaments in resting *Trpm7*^*fl/fl-Pf4Cre*^ platelets (Supplementary Fig. 9), sedimentation of cross-linked actin filaments of resting platelets revealed an increased amount of NMMIIA in the pellet fraction (Supplementary Fig. 11a–c). On activation, NMMIIA efficiently cross-linked actin filaments and accumulated in the pellet fraction of WT platelets (Supplementary Fig. 11d–f). In sharp contrast, we found a marked reduction in NMMIIA on stimulation of *Trpm7*^*fl/fl-Pf4Cre*^ platelets (Supplementary Fig. 11d–f), similar to the findings in spread MKs (Fig. 3c) or platelets (Supplementary Fig. 10a–d). To exclude that the observed effects were due to the short observation period, bone marrow-derived MKs were cultured for 48 h in the presence of collagen types I or IV, two major components of the extracellular matrix in the bone marrow. Strikingly, we found a reduced content of NMMIIA in *Trpm7*^*fl/fl-Pf4Cre*^ MKs (reduction of 39.9±10.3% for collagen I and 76.9±14.5% for collagen IV) cultured in the presence of different collagens as compared with control cells (Fig. 3h; Supplementary Fig. 12a,b). Together these results suggested an increased NMMIIA activity under resting conditions and that NMMIIA undergoes a rapid degradation on stimulation of platelets and MKs from *Trpm7*^*fl/fl-Pf4Cre*^ mice thus allowing proplatelet formation and cell spreading.

### Deregulated [Mg^2+^]_i_ perturbs NMMIIA activity

**Figure 4:**
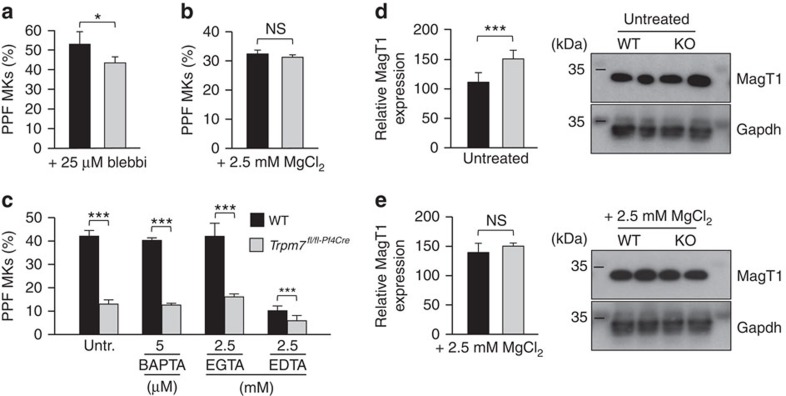
Altered NMMIIA regulation accounts for the impaired proplatelet formation. (**a**–**c**) Pretreatment (90 min) of foetal liver-derived MKs with 25 μM blebbistatin (**a**) or 2.5 mM MgCl_2_ (**b**) rescued proplatelet formation of *Trpm7*^*fl/fl-Pf4Cre*^ MKs and increased those of WT controls. (**c**) Pretreatment (90 min) with the Ca^2+^ chelators BAPTA-AM or EGTA (ethylene glycol-bis(2-aminoethylether)-*N,N,N′,N′*-tetraacetic acid) did not interfere with proplatelet formation whereas the non-selective cation chelator EDTA strongly impaired proplatelet formation in both WT and *Trpm7*^*fl/fl-Pf4Cre*^ foetal liver-derived MKs. Values are mean±s.d. (*n*=6). (**d**,**e**) Densitometric analyses on immunoblots reveal a compensatory upregulation of MagT1 expression under normal culture conditions (**d**) in *Trpm7*^*fl/fl-Pf4Cre*^ MKs that can be restored to the expression levels of WT controls by Mg^2+^ supplementation (**e**). All images are representative of at least five animals. Values are mean±s.d. (*n*=5). Unpaired Student's *t*-test; ***P<0.001; *P<0.05; NS, non-significant.

We hypothesized that deregulated Mg^2+^ homeostasis^26^ and increased NMMIIA activity may account for the impaired proplatelet formation in *Trpm7*^*fl/fl-Pf4Cre*^ MKs (refs 16, 26). In support of this, either inhibition of NMMIIA activity or Mg^2+^ supplementation could almost fully restore proplatelet formation of *Trpm7*^*fl/fl-Pf4Cre*^ MKs *in vitro* (Fig. 4a,b). Of note, while pretreatment of foetal liver- (Fig. 4c) or bone marrow-derived MKs (Supplementary Fig. 13) with the Ca^2+^ chelators 1,2-bis(2-aminophenoxy) ethane-N,N,N′,N′-tetraacetic acid tetrakis(acetoxymethyl ester) (BAPTA-AM) or ethylene glycol tetraacetic acid (EGTA) did not exert gross effects; non-specific chelation of Ca^2+^ and Mg^2+^ with ethylenediaminetetraacetic acid (EDTA) significantly reduced proplatelet formation by MKs, suggesting that Mg^2+^ is critical for this process^28^,^29^. According to a previous report^8^, Mg^2+^ supplementation should restore [Mg^2+^]_i_ in mutant MKs, which cannot be fully achieved through an upregulation of *MagT1* expression under normal culture conditions (Fig. 4d,e).

### Inhibition of NMMIIA restores cytoskeletal architecture

**Figure 5:**
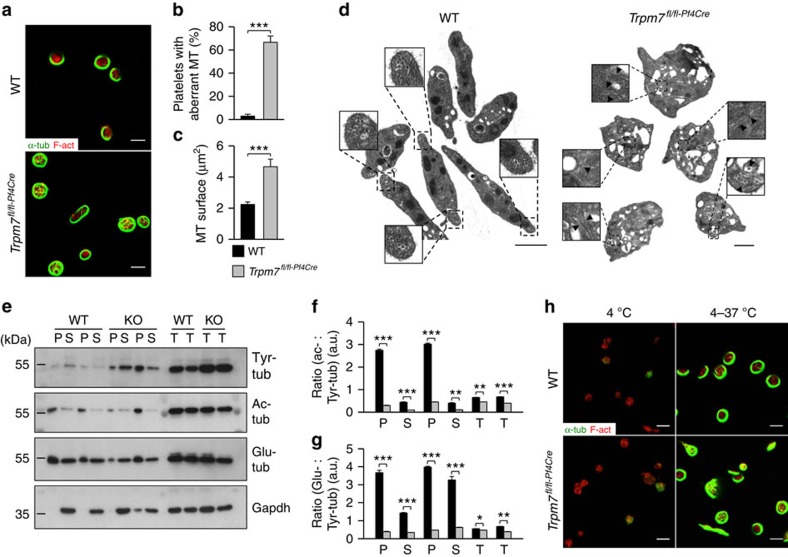
Altered cytoskeletal organization in *Trpm7*^*fl/fl-Pf4Cre*^ platelets. (**a**) Confocal images of resting platelets. The platelet cytoskeleton was stained for α-tubulin (green) and F-actin (red). Scale bars, 3 μm. (**b**,**c**) Quantification of platelets with aberrant (**b**) microtubules (MT) and the MT surface per platelet (**c**). Values are mean±s.d. (*n*=5; 200 platelets). (**d**) Transmission electron microscopy (TEM) analysis of resting *Trpm7*^*fl/fl-Pf4Cre*^ platelets revealed an anarchic organization of microtubules (arrow heads in inlays). Scale bar, 1 μm. (**e**) Tubulin cytoskeleton of resting platelets was isolated by ultracentrifugation of Triton X-100 lysates and immunoblotted to detect dynamic Tyr-tubulin and post-translational modifications of the microtubules by the analysis of acetylated (ac)- or detyrosinated (Glu)-tubulin. Gapdh served as loading control. Insoluble fraction (pellet, P); soluble fraction (supernatant, S); total protein (T). (**f**,**g**) Densitometry revealed a markedly reduced ratio of stable acetylated (**f**) and detyrosinated (**g**) microtubules to highly dynamic Tyr microtubules in *Trpm7*^*fl/fl-Pf4Cre*^ platelets. Values are mean±s.d. (*n*=6) (**h**) Platelets were incubated for 3.5 h at 4 °C and if indicated rewarmed at 37 °C, fixed on PLL-coated slides and stained for F-actin (red) and α-tubulin (green). All images are representative of at least five animals. Unpaired Student's *t*-test; ***P<0.001; **P<0.01; *P<0.05.

**Figure 6:**
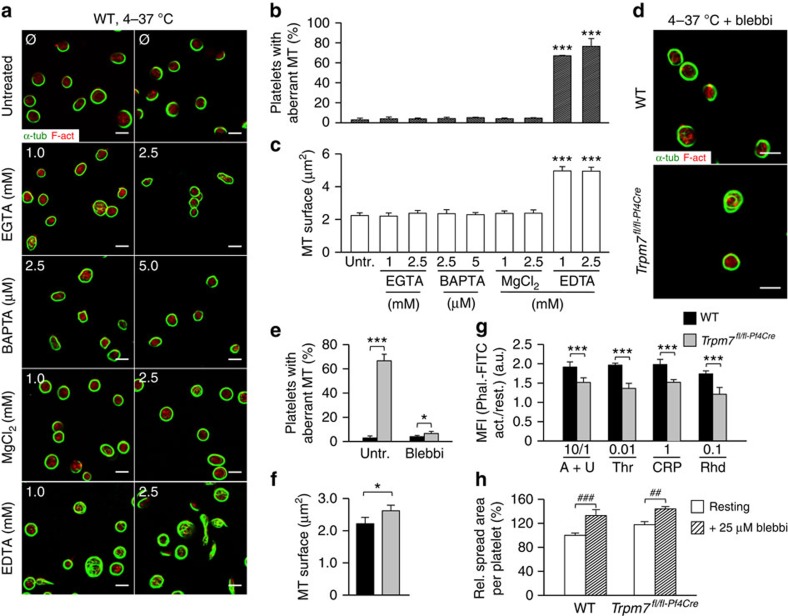
Deregulated [Mg^2+^]_i_ accounts for the altered cytoskeletal organization. (**a**) Resting control platelets were incubated for 3 h at 4 °C in Tyrode's-HEPES buffer supplemented with the indicated reagents. After rewarming to 37 °C platelets were fixed, allowed to adhere to PLL-coated coverslips and were stained for α-tubulin (green) and F-actin (red). (**b**,**c**) Quantification of microtubule (MT) morphology (**b**) and surface (**c**) revealed that decreasing [Mg^2+^]_i_ (EDTA) but not [Ca^2+^]_i_ (EGTA or BAPTA) in WT platelets reproduces the cytoskeletal alterations found in *Trpm7*^*fl/fl-Pf4Cre*^ platelets. Values are mean±s.d. (*n*=5; 200 platelets). (**d**–**f**) Rewarming of chilled and blebbistatin-pretreated (25 μM) *Trpm7*^*fl/fl-Pf4Cre*^ platelets restored cytoskeletal organization. Scale bars, 3 μm. Values are mean±s.d. (*n*=6; 200 platelets). (**g**) The ratio of polymerized actin in activated versus resting platelets was determined. Platelet stimulation was achieved using A+U, 10 μM ADP and 1 μM U46619; Thr, 0.01 U ml^−1^ thrombin; CRP, 1 μg ml^−1^ collagen-related peptide; Rhd, 0.1 μg ml^−1^ rhodocytin. Values are mean±s.d. (*n*=6). (**h**) The relative spread surface area of untreated or blebbistatin-treated (25 μM) platelets was determined using F-actin staining as a measure. Values are mean±s.d. (*n*=6; 200 platelets). All images are representative of at least five animals. Unpaired Student's *t*-test; ***^,###^*P*<0.001; ^##^<*P*<0.01; *P<0.05.

Besides increasing cortical tension through relaxation of the underlying cytoskeleton^17^,^30^, blebbistatin treatment reduced the prevalence and stability of microtubules in proplatelet protrusions, as evidenced by decreased Glu-/Tyr- (1.1±0.1 for WT and 1.3±0.1 for samples of *Trpm7*^*fl/fl-Pf4Cre*^ mice) and ac-/Tyr-tubulin ratios (1.3±0.1 for WT and 1.2±0.2 for samples of *Trpm7*^*fl/fl-Pf4Cre*^ mice; Supplementary Fig. 14). Immunostaining (Fig. 5a–c) and electron microscopy (Fig. 5d) revealed an increased number of aberrantly organized microtubules in *Trpm7*^*fl/fl-Pf4Cre*^ platelets as compared with controls, similar to findings in mutant MKs. This correlated with an increased presence of highly dynamic Tyr-tubulin (151.4±3.7% of controls; Fig. 5e–g), leading to accelerated and uncontrolled microtubule polymerization in mutant cells (Fig. 5a,h). Stable microtubules in *Trpm7*^*fl/fl-Pf4Cre*^ platelets displayed a similar pattern of post-translational modifications of α-tubulin (104.2±14.2% of control for Glu-, and 97.4±9.4% of control for ac-tubulin) and were efficiently disassembled on cold challenge, thus excluding enhanced stability as cause for the increased microtubule content (Fig. 5e–h). Interestingly, pretreatment of WT platelets with EDTA, but not EGTA, BAPTA-AM or MgCl_2_, mimicked these cytoskeletal alterations (Fig. 6a–c), changes that could also be reverted by blebbistatin (Fig. 6d–f), thus further supporting the notion that reduced [Mg^2+^]_i_ alters the subcellular localization and activity of NMMIIA resulting in cytoskeletal disorganization. In agreement with this, we observed an increased content of filamentous actin in resting *Trpm7*^*fl/fl-Pf4Cre*^ platelets (Supplementary Fig. 15a,b) and a decreased polymerization of filamentous actin on platelet activation (Fig. 6g; Supplementary Fig. 15b,c). Moreover, spread *Trpm7*^*fl/fl-Pf4Cre*^ platelets displayed an increased surface area (Fig. 6h; Supplementary Fig. 16) most likely reflecting the activation-dependent rapid degradation of NMMIIA and consequently the loss of coherent cytoplasmic contractile forces normally generated by activated NMMIIA (refs 31, 32).

### Variants in *TRPM7* may cause macrothrombocytopenia

We next hypothesized that some DNA variants of extreme low-frequency affecting TRPM7 channel function might also cause macrothrombocytopenia in humans. Examining the results of genome sequencing of 702 cases with bleeding and platelet disorders of unknown genetic basis in the BRIDGE database of the NIHR BioResource—Rare Diseases revealed three cases with a coding variant unobserved in nearly 81,000 control DNA samples: UCN 0012 with p.C721G (c.2161T>G), UCN 0025 with p.R902C (c.2704C>T) variant, and UCN 0110 with p.G1353D (c.4058G>A; Supplementary Table 1). Two of these three index cases (p.C721G and p.R902C) showed low-platelet counts and macrothrombocytopenia. Interestingly, the variant of index patient UCN 0110 (p.G1353D (c.4058G>A)) was located close to the α-kinase domain and the absence of macrothrombocytopenia is in agreement with the results on the *Trpm7*^*KI*^ mice (Supplementary Table 1 and Supplementary Fig. 2). Unfortunately, the family with the p.R902C variant was unavailable for further studies.

**Figure 7:**
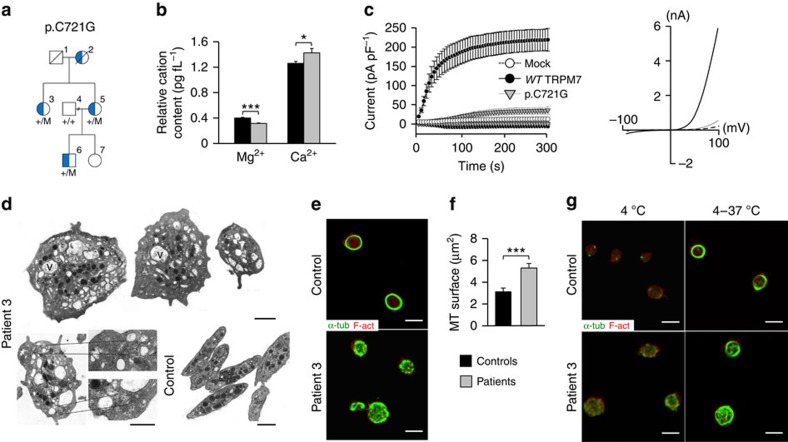
A human genetic variant in *TRPM7* causes macrothrombocytopenia. (**a**) A heterozygous p.C721G variant of *TRPM7* was identified by whole-exome sequencing in an index patient. Sanger sequencing confirmed that this variant (M) cosegregated with the macrothrombocytopenia (blue coloration) in the family pedigree. Open symbols indicate that no macrothrombocytopenia was observed. +/M indicates that the family member was a carrier of the *TRPM7* variant. +/+ indicates that no variant was detected at that locus and no symbol that genotyping was not performed. (**b**) Total platelet cation content of healthy controls and patients 3, 5 and 6 was determined by inductively coupled mass spectrometry. Values are mean±s.d. (*n*=5 controls versus 3 patients). (**c**) Whole-cell patch clamp measurements on mock-transfected HEK293 (*n*=8), and cells overexpressing WT TRPM7 (*n*=13) or the p.C721G variant (*n*=13) revealed impaired channel activity. Measurements were conducted in absence of extracellular Mg^2+^ to enhance current sizes. Currents were elicited by a ramp protocol from −100 to +100 mV over 50 ms acquired at 0.5 Hz. Left panel: inward current amplitudes were extracted at −80 mV, outward currents at +80 mV and plotted versus time of the experiment. Values are normalized to cell size as pA pF^−1^ and represent mean±s.e.m. The depletion of intracellular Mg^2+^ leads to the development of characteristic TRPM7-like currents in WT TRPM7 overexpressing HEK293 cells, whereas TRPM7 currents were abolished in mock-transfected, or p.C721G overexpressing HEK293 cells. Right panel: representative current–voltage relationships extracted at 250 s. WT TRPM7 overexpressing HEK293 cells show an I V^−1^-relationship characteristic for TRPM7 (black trace), which are absent in mock-transfected (dashed black trace) or p.C721G overexpressing HEK293 cells (light grey trace). (**d**) Transmission electron microscopy (TEM) analysis of platelets reveals the abnormal platelet morphology associated with the mutation. V, vacuole. Scale bars, 1 μm. (**e**–**g**) Poly-L-lysine-immobilised resting (**e**) or cold-challenged (**g**) platelets were permeabilised and stained for F-actin (red) α-tubulin (green) and analysed by confocal microscopy. (**f**) Image analysis revealed an increased prevalence of microtubules (MT) under resting conditions. Values are mean±s.d. (*n*=3; 200 platelets). Unpaired Student's *t*-test; ***P<0.001; *P<0.05.

Further studies were focussed on the index case UCN 0012 with a p.C721G (c.2161T>G) variant and pedigree members. Sanger sequencing showed that the p.C721G variant was present in two further pedigree members with macrothrombocytopenia, but was absent in one asymptomatic pedigree member, indicating segregation with the *TRPM7* genotype (Fig. 7a, Supplementary Table 2 and Supplementary Fig. 17). For the fourth patient (pedigree member 2), now deceased, macrothrombocytopenia was detected during her life (Fig. 7a). All other blood cell parameters and platelet function were normal for all p.C721G patients. Strikingly, paroxysmal atrial fibrillation was also present for the index case (pedigree member 5) and her mother (pedigree member 2).

Similarly to *Trpm7*^*fl/fl-Pf4Cre*^ mouse platelets, we found a reduced content of Mg^2+^ and an increased concentration of Ca^2+^ in platelets from all tested patients with the p.C721G substitution as compared with healthy controls (Fig. 7b). Patch clamp studies on HEK293 cells confirmed that the p.C721G variant reduced TRPM7 channel activity by 85±4% as compared with WT controls (Fig. 7c) despite being localized to the cell membrane (Supplementary Fig. 18). Likewise, *in vitro* studies on the p.R902C variant revealed, although less pronounced, a reduced TRPM7 channel activity by 39±6% (Supplementary Fig. 19). Platelets from all tested patients displayed an increased size, with a spherical shape and contained numerous vacuoles. Moreover, while platelets from controls displayed the typical discoid shape and microtubules organized into coils, the marginal band, platelets from the p.C721G patients showed an aberrant distribution of granules and an increased number and anarchic organization of microtubules (Fig. 7d; Supplementary Fig. 20). The abnormal cytoskeletal organization could be confirmed by immunostaining on resting p.C721G platelets (Fig. 7e,f; Supplementary Figs 21 and 22) and similarly to the *Trpm7*^*fl/fl-Pf4Cre*^ platelets, which was not associated with increased microtubule stability (Fig. 7g; Supplementary Fig. 21).

### Altered regulation of NMMIIA in p.C721G platelets

**Figure 8:**
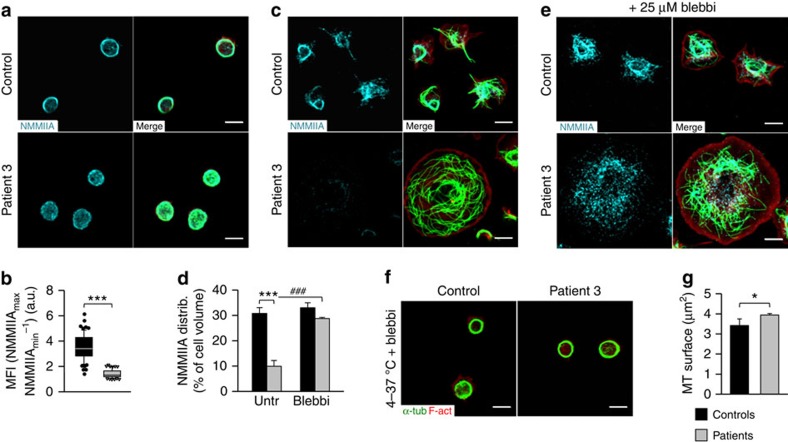
Altered NMMIIA activity accounts for the aberrant cytoskeletal organization. (**a**) Poly-L-lysine-immobilised resting platelets were permeabilized and stained for F-actin (red) α-tubulin (green), NMMIIA (cyan) and analysed by confocal microscopy. (**b**) Image analysis revealed an altered distribution of NMMIIA in platelets from patients with variants in *TRPM7* as compared with healthy controls. Box plots display first and third quartiles and whiskers mark minimum and maximum values unless exceeding 1.5 × interquartile range (IQR) of at least 70 platelets; symbols represent outliers and the horizontal line displays median (*n*=3 controls versus patient 3). Wilcoxon–Mann–Whitney test; ***P<0.001 (**c**–**e**) Degradation of NMMIIA on spreading of platelets from patient 3 (**c**,**d**) could be prevented by blebbistatin pretreatment (**d**,**e**). Values are mean±s.d. (*n*=3; 200 platelets). (**f**,**g**) Pretreatment of resting platelets from patient 3 with 25 μM blebbistatin restored cytoskeletal organization on cold challenge with subsequent rewarming (**f**), which was further evidenced by a significantly decreased surface covered by microtubules (MT) per platelet (**g**). Values are mean±s.d. (*n*=3; 200 platelets). Unpaired Student's *t*-test (if not stated otherwise); ***^,###^*P*<0.001; *P<0.05.

Since the ultrastructure of human platelets from individuals carrying the p.C721G variant strongly resembled that of *Trpm7*^*fl/fl-Pf4Cre*^ mouse platelets, we next analysed the distribution and stability of NMMIIA. Whereas in control platelets NMMIIA localized to the marginal band, it was homogeneously distributed throughout the cytoplasm of platelets from the patients (Fig. 8a,b; Supplementary Fig. 22). In line with the observations on *Trpm7*^*fl/fl-Pf4Cre*^ mouse platelets, spread platelets from the patients showed an increased surface area, a strong decrease in NMMIIA and an increased content of microtubules (Fig. 8c,d; Supplementary Figs 23 and 24). As for the mouse model, blebbistatin prevented loss of NMMIIA on spreading (Fig. 8d,e; Supplementary Fig. 25) and rescued the cytoskeletal organization of resting platelets after cold challenge (Fig. 8f,g; Supplementary Fig. 26). As mentioned above, patients 2 and 5 suffered from atrial fibrillation which might be associated with alterations in [Mg^2+^]_i_ (ref. 33). Furthermore, lack of TRPM7 has been associated with altered expression of channels, such as HCN4 encoding the pacemaker current in the conduction system of the heart that has also previously been linked to atrial fibrillation^20^,^34^.

## Discussion

In the present study, we provide evidence that defects in TRPM7 channel function cause macrothrombocytopenia in mice and likely in humans too. These results demonstrate a critical role of TRPM7-mediated Mg^2+^ influx in regulating NMMIIA activity and cytoskeletal rearrangements during thrombopoiesis. In support of this, it has been shown that Mg^2+^ inhibits NMMIIA activity by reducing the ADP release rate and its affinity for actin^26^. Furthermore, using *Trpm7*^*KI*^ mice (Supplementary Fig. 2), we convincingly show that these effects occur independently of TRPM7 α-kinase activity. However, it is too early to exclude that the kinase domain also acts as a molecular hub orchestrating yet unknown signalling events, and thereby contributes to the development of macrothrombocytopenia.

The observed loss of NMMIIA in *Trpm7*^*fl/fl-Pf4Cre*^ bone marrow MKs *in situ* and *in vitro* on spreading could represent a physiological process to overcome the inhibitory effects of increased NMMIIA activity enabling proplatelet formation (Fig. 3a,b,d–h; Supplementary Figs 10–12). In support of this hypothesis, NMMIIA degradation was most pronounced on culture of bone marrow-derived MKs on collagen type IV, which predominates in the vascular niche where proplatelet formation occurs (Fig. 3a,b,h; Supplementary Fig. 12). Furthermore, using different antibodies and experimental approaches, we provide several independent lines of evidence that the observed loss of NMMIIA on activation or spreading of TRPM7-deficient cells represents protein degradation. However, it is important to note that we cannot entirely exclude epitope masking due to modifications on NMMIIA (Figs 3 and 8; Supplementary Figs 10–12 and 23–25).

It has recently been reported that plasma Thpo levels are also regulated via Ashwell–Morell receptor-dependent clearance of desialylated platelets, which in turn triggers expression of *Thpo* in hepatocytes and thus regulates platelet production^35^. However, it is important to note that in agreement with the moderately reduced platelet lifespan, we did not observe differences in platelet terminal galactose levels suggesting that the decreased plasma Thpo levels are reciprocally associated to the increased number of MKs in the bone marrow and spleen (Fig. 2a,b; Supplementary Figs 3 and 4)^36^. Furthermore, the marked increase in platelet counts after immuno-depletion might result from an alternative platelet release mechanism as recently described by Nishimura *et al*.^25^.

The here-described human disorder (Fig. 7; Supplementary Tables 1 and 2) is clearly distinguishable from *MYH9*-related platelet disease, which can be associated with hearing loss, cataracts or renal failure^37^. However, patients with variants in *TRPM7* and *MYH9* both display macrothrombocytopenia with more spherical platelets and partially, increased actomyosin contractility (Figs 7 and 8; Supplementary Figs 21–26)^16^. The different clinical outcomes and symptoms of patients with different genetic variants of *TRPM7* are most likely due to the location and functional consequences of the mutations (Supplementary Table 1). While both the p.C721G and the p.R902C variant are located in the channel domain and reduce channel activity (Fig. 7c and Supplementary Fig. 19), the p.G1353D variant is not located within the channel domain which may likely explain the distinct phenotype and absence of macrothrombocytopenia in the index patient UCN 0025. Similar genotype–phenotype-relationships have been observed for patients suffering from *MYH9*-related disorder^38^,^39^, Wiskott–Aldrich syndrome^2^,^40^ or filaminopathy A^41^. Although we could not observe a dominant negative effect of the p.C721G variant on TRPM7 channel function *in vitro*, we speculate that the defect might be masked by the high-expression levels of TRPM7 in the used system or diminished channel activity may not represent the only mechanistic explanation for the observed phenotypes. Furthermore, *Trpm7*^*+/*−^ mice did not display macrothrombocytopenia suggesting that gene haploinsufficiency does not account for the observed disease (Supplementary Fig. 27). However, the impaired channel activity and the striking phenotypic similarities in platelets from patients and *Trpm7*^*fl/fl-Pf4Cre*^ mice strongly suggest that the variants in *TRPM7* account for the observed macrothrombocytopenia.

Our study provides for the first time several independent lines of evidence that proper regulation of Mg^2+^ homeostasis in MKs by TRPM7 plays a critical role in thrombopoiesis and platelet sizing, both in humans and mice. Collectively, our results highlight the clinical need to carefully control platelet count and size in patients with deregulated Mg^2+^ homeostasis. Ultimately, our findings suggest, after careful consideration and assessment of adverse events, that Mg^2+^ supplementation may be used as a potential treatment of patients with increased activity of NMMIIA to manage thrombocytopenia. Despite the possibility of severe potential side effects in patients with impaired kidney function, Mg^2+^ supplementation is considered as a relatively safe therapeutic intervention^42^,^43^,^44^. Nevertheless, further studies in animal models and patients are required to assess the efficacy and safety of Mg^2+^ supplementation in the management of certain cases of thrombocytopenia.

Finally, the fact that two of the patients in our study also suffered from paroxysmal atrial fibrillation promotes *TRPM7* as a novel candidate interfering with conductance in cardiac cells.

## Methods

### Semi-quantitative reverse transcription PCR

Total platelet RNA was isolated after lysis using TRIzol reagent (15596018, Invitrogen) and tissue RNA was gained using the Qiagen RNeasy kit. To generate cDNA 1 μg RNA was reverse transcribed with the SuperScript II reverse transcriptase (18064014, Invitrogen) according to the manufacturer's instructions. β-actin transcripts were determined as control. Primers used for the amplification of Mg^2+^ transporters and channels are listed in Supplementary Table 3.

### Mice

Conditional Trpm7-deficient mice were generated by intercrossing *Trpm7*^*fl/fl*^ mice (exon 17 flanked by loxP sites) with mice carrying the Cre-recombinase under the platelet factor 4 (*Pf4*) promoter^45^ (Supplementary Fig. 1b). *Trpm7*^*fl/fl*^ mice were described earlier^4^, *Pf4-Cre* mice were kindly provided by Dr Radek Skoda and *Trpm7*^*KI*^ mice were generously provided by Dr Masayuki Matsushita^6^. All mice used in experiments were 12- to 16-week-old and sex-matched, if not stated otherwise. For experiments on MKs, only male animals were used. All animal experiments were approved by the district government of Lower Franconia (Bezirksregierung Unterfranken). *Trpm7*^*fl/fl-Pf4Cre*^ mice were genotyped via PCR on genomic DNA extracted from ear biopsies using the following primer pair: Trpm7KO-F: 5′-gaggtactggcaattgtgagc-3′ and Trpm7KO-R: 5′-accacaaaatctctgccctct-3′ yielding a 1,200-bp product for the floxed, 1000, bp product for the WT and a 400-bp fragment for the recombined allele (Supplementary Fig. 1c). For all experiments, the respective *Trpm7*^*fl/fl*^ (for *Trpm7*^*fl/fl-Pf4Cre*^ mice) or *Trpm7*^*WT/WT*^ (for *Trpm7*^*KI/KI*^ mice) littermate controls were used. All mice were derived from the following breeding strategies for *Trpm7*^*fl/fl*^ X *Trpm7*^*fl/fl-Pf4Cre*^, yielding 50% *Trpm7*^*fl/fl*^ WT and 50% *Trpm7*^*fl/fl-Pf4Cre*^ mice or *Trpm7*^*WT/KI*^ X *Trpm7*^*WT/KI*^ resulting in 25% *Trpm7*^*WT/WT*^, 50% *Trpm7*^*WT/KI*^ and 25% *Trpm7*^*KI/KI*^ mice, respectively.

### Platelet preparation

Mice were bled under isoflurane anaesthesia. Blood was collected in heparin (20 U ml^−1^, Ratiopharm) and centrifuged twice for 6 min at 300 g. Platelet-rich plasma (PRP) was supplemented with 2 μl ml^−1^ of apyrase (0.02 U ml^−1^; A6410, Sigma-Aldrich) and 5 μl ml^−1^ prostacyclin I2 (PGI_2_) (0.1 μg ml^−1^; P6188, Sigma-Aldrich) and platelets were pelleted by centrifugation for 5 min at 800 g, washed twice with Tyrodes-N-2-hydroxyethyl-piperazine-N′2-ethanesulfonic acid (HEPES) buffer (134 mM NaCl, 0.34 mM Na_2_HPO_4_, 2.9 mM KCl, 12 mM NaHCO_3_, 5 mM HEPES, 5 mM glucose, 0.35% bovine serum albumin (BSA), pH 7.4) containing 2 μl ml^−1^ apyrase and 5 μl ml^−1^ PGI_2_.

Blood samples of patients were obtained after informed consent in accordance with the Declaration of Helsinki. Ethical approval was obtained from Inserm RBM 04–14 for the project ‘Network on the inherited diseases of platelet function and platelet production' and by the Ethics Committee of the University Hospital Würzburg. Fresh blood samples of patients and healthy volunteers were collected in 1/10 volume of acid-citrate-dextrose and centrifuged for 10 min at 200 g. PRP was collected, supplemented with 2 μl of apyrase (0.02 U ml^−1^, Sigma-Aldrich) and 5 μl PGI_2_ (0.1 μg ml^−1^, Sigma-Aldrich) per ml PRP. Before adherence to poly-L-lysine (PLL)-coated slides or spreading on different matrices, platelets were pelleted by centrifugation for 10 min at 800 g and washed twice with Tyrodes-HEPES buffer containing 2 μl ml^−1^ apyrase and 5 μl ml^−1^ PGI_2_. The samples were allowed to rest for 30 min at 37 °C prior to being used in experiments.

### Flow cytometry

Diluted blood (1:20) was incubated for 15 min at room temperature (RT) with fluorophore-conjugated antibodies (2 μg ml^−1^) directed against platelet surface glycoproteins. Platelet count and size (FSC) were assessed using a FACSCalibur (BD Biosciences) flow cytometer or with an automated blood cell analyser (Sysmex KX-21N).

### Transmission electron microscopy of platelets

To analyse platelet ultrastructure, washed platelets were fixed with 2.5% glutaraldehyde (16,210, Electron Microscopy Sciences) in 50 mM cacodylate buffer (12,201, pH 7.2; AppliChem). After embedding in epon 812 (14,900, Electron Microscopy Sciences), ultrathin sections were generated and stained with 2% uranyl acetate (22,400, Electron Microscopy Sciences) and lead citrate (17,800, Electron Microscopy Sciences). Samples were visualized with an EM900 transmission electron microscope (Carl Zeiss).

### Immunostaining on resting or spread platelets

Coverslips were either coated with PLL (Sigma-Aldrich), fibrinogen (100 μg ml^−1^; F4883, Sigma-Aldrich) or collagen-related peptide (CRP) (ref. 46; 6 μg ml^−1^) overnight at 4 °C. For spreading on fibrinogen, platelets were stimulated with 0.01 U ml^−1^ thrombin (10602400001, Roche) and allowed to spread for the indicated time. If indicated, platelets were pre-incubated with toxins interfering with cytoskeletal dynamics such as colchicine (10 μM; A4082, AppliChem) before the spreading assay. After the indicated time points, platelets were either fixed with 4% paraformaldehyde (PFA) in PBS and analysed or processed for immunofluorescence staining of the cytoskeleton. Therefore, platelets were fixed and permeabilized in PHEM buffer (60 mM piperazine-N,N-bis-2- ethanesulfonic acid, 25 mM HEPES), 10 mM EGTA, 2 mM MgCl_2_, pH 6.9) supplemented with 4% PFA and 1% IGEPAL CA-630, stained with anti-α-tubulin-Alexa F488 (3.33 μg ml^−1^, 322588 (B-5-1-2), Invitrogen), anti-NMMIIA (10 μg ml^−1^, #3403, polyclonal, CellSignaling) and phalloidin-Atto647N (170 nM, 65906, Fluka) and mounted with Fluoroshield (F6182, Sigma-Aldrich). All used fluorophore-conjugated secondary antibodies were purchased from Invitrogen. Samples were visualized using a Leica TCS SP5 confocal microscope (Leica Microsystems).

### Inductively coupled plasma mass spectrometry (ICP-MS)

The cation content of 4 × 10^7^ platelets was analysed by ICP-MS on platelets from PRP. ICP-MS analysis was performed by ALS Scandinavia AB (Lulea, Sweden).

### Determination of platelet lifespan

The clearance of platelets from the circulation was determined by the retro-orbital injection of 5 μg DyLight 488-labelled anti-GPIX antibody^2^,^47^ in PBS into male mice. The percentage of labelled platelets was determined by daily blood withdrawal and subsequent analysis by flow cytometry using fluorophore-conjugated platelet-specific antibodies^2^.

### Immunofluorescence staining on whole-femora cryosections

Femora of male mice were isolated, fixed with 4% paraformaldehyde (A3813, AppliChem) and 5 mM sucrose (S0389, Sigma-Aldrich), transferred into 10% sucrose in PBS and dehydrated using a graded sucrose series. Subsequently the samples were embedded in Cryo-Gel (39475237, Leica Biosystems) and shock frozen in liquid nitrogen. Frozen samples were stored at −80 °C. Cryosections (7-μm-thick) were generated using the CryoJane tape transfer system (Leica Biosystems) and probed with Alexa488-conjugated anti-glycoprotein (GP) Ib (ref. 48) antibodies (7A9, 1.33 μg ml^−1^), to specifically label platelets and MKs, and Alexa647-conjugated anti-CD105 antibodies (3.33 μg ml^−1^, 120402 (MJ7/18), Biolegend) to stain the endothelium. Nuclei were stained using 4′,6-diamidino-2-phenylindole (1 μg ml^−1^, D1306, Invitrogen). Samples were visualized with a Leica TCS SP5 confocal microscope (Leica Microsystems).

### Histology

Sections (3-μm-thick) of formalin-fixed paraffin embedded spleens and femora of male mice were prepared deparaffinized and stained with haematoxylin (MHS32, Sigma-Aldrich) and eosin (318906, Sigma-Aldrich). MK number, morphology and localization were analysed with an inverted Leica DMI 4000 B microscope.

### *In vitro* differentiation and cultivation of bone marrow MKs

Haematopoietic stem cells were isolated from male mouse bone marrow single-cell suspensions using a magnetic bead-based negative depletion kit (anti-rat-IgG Dynabeads, Invitrogen) in combination with rat-anti-mouse antibodies directed against CD45R/B220, TER-119, CD3, Ly-6G/C and CD11b (each 0.5 μg per 10^7^ cells, 103216 (RA3-6B2), 116214 (TER-119), 100208 (17A2), 108414 (RB6-8C5) and 101214 (M1/70), Biolegend). Experiments were performed according to the manufacturer's protocol. Cells were cultured in MK-medium supplemented with 50 μg ml^−1^ recombinant hirudin (Schering) at 37 °C, 5% CO_2_ for 3 days, before MK enrichment using a two step BSA density gradient. On day 4, the percentage of proplatelet-forming MKs was determined using a light microscope (Zeiss).

### MK spreading

*In vitro* cultivated bone marrow MKs of male mice were allowed to adhere and spread at 37 °C and 5% CO_2_ on coverslips coated with fibrillar collagen type I (50 μg ml^−1^; Nycomed), fibrinogen (100 μg ml^−1^; F4883, Sigma-Aldrich) or CRP (6 μg ml^−1^) for the indicated time. MK spreading was stopped by fixation and permeabilization of the cells using PHEM buffer supplemented with 4% PFA and 1% IGEPAL CA-630.

### Immunofluorescence staining of cultured or spread MKs

To visualize the cytoskeleton, the cultured MKs were spun onto glass slides (Shandon Cytospin 4, Thermo Scientific), fixed and permeabilized in PHEM buffer supplemented with 4% PFA and 1% IGEPAL CA-630 and blocked with 1% BSA in PBS. F-actin was stained using phalloidin-Atto647N (170 nM, 65906, Fluka) and tubulin was stained with an anti-α-tubulin-Alexa F488 (3.33 μg ml^−1^, 322588 (B-5-1-2), Invitrogen) antibody. WASp (10 μg μl^−1^, #4860, CellSignaling) or NMMIIA (10 μg ml^−1^, #3403, polyclonal, CellSignaling) staining served as podosome marker. Nuclei were stained using 4′,6-diamidino-2-phenylindole (1 μg μl^−1^, D1306, Invitrogen) before mounting of samples with Fluoroshield (F6182, Sigma-Aldrich). Visualization was performed with a Leica TCS SP5 confocal microscope (Leica Microsystems).

### Determination of MK ploidy

To determine bone marrow MK ploidy, both femora of male mice were isolated and the bone marrow was flushed and homogenized. Non-specific binding sites of the 5D7 antibody were blocked by incubation of the cell suspension with 0.02 μg μl^−1^ anti-FcγR antibody (553142 (2.4G2), BD Pharmingen). Afterwards, MKs were stained using a fluorescein isothiocyanate-conjugated anti-CD41 antibody (10 μg ml^−1^, 5D7). Finally, cells were fixed, permeabilized and DNA was stained using 50 μg ml^−1^ propidium iodide (P3561, Invitrogen) staining solution with 100 μg ml^−1^ RNaseA (EN0202, Fermentas) in PBS. Analysis was performed by flow cytometry and FlowJo software (Tree Star Inc.).

### Plasma thrombopoietin levels

Plasma Thpo levels were determined using the Mouse Thrombopoietin Quantikine ELISA Kit (DY488, R&D Systems). Briefly, plasma was collected, diluted (1:5 in Reagent Diluent) and immediately applied as duplicates onto the anti-Thpo-IgG coated 96-well plate and incubated for 2 h at room temperature. The plate was washed five times and incubated for 2 h with 100 μl of horseradish peroxidase-conjugated anti-mouse Thpo antibodies. After another five washing steps, tetramethylbenzidine solution was added and incubated for 30 min at room temperature. The reaction was aborted by the addition of 100 μl of diluted hydrochloric acid. Optical densities of the samples were determined using a Multiskan Ascent (96/384) plate reader (MTX Lab Systems) at 450 nm. Wavelength correction was performed at 570 nm.

### *In vitro* differentiation of foetal liver-derived MKs

The livers of 13.5–14.5-day-old female and male mouse foetuses were isolated from time-mated female mice and single-cell suspensions were prepared in MK-medium (IMDM medium containing 10% FCS, 1% penicillin/streptomycin (31980097, Gibco) and 50 ng ml^−1^ recombinant Thpo (ref. 49). The homogenized foetal liver cells were cultured for 72 h at 37 °C and 5% CO_2_ and mature MKs were enriched on day three of culturing using a BSA density gradient (3% and 1.5% BSA in PBS; A7030, Sigma-Aldrich). If indicated the cells were treated with 25 μM blebbistatin (B0560, Sigma-Aldrich) or maintained in culture medium supplemented with 10 mM MgCl_2_. On day 4, the percentage of proplatelet-forming MKs was determined by counting the total number of MKs as well as the number of proplatelet-forming MKs under a light microscope (Zeiss).

### Two-photon *intravital* microscopy of the bone marrow

Male and female mice were anaesthetized and a 1-cm incision was made along the midline to expose the frontoparietal skull while carefully avoiding damage to the bone tissue. The mouse was placed on a customized metal stage equipped with a stereotactic holder to immobilize its head^2^,^3^. Bone marrow vasculature was visualized by injection of tetramethylrhodamine dextran (8 μg per g body weight, 2 MDa, Molecular Probes). Platelets and MKs were antibody-stained (0.6 μg per g body weight anti-GPIX-Alexa Fluor 488). Images were acquired with a fluorescence microscope equipped with a × 20 water objective with a numerical aperture of 0.95 and a TriM Scope II multiphoton system (LaVision BioTec), controlled by ImSpector Pro-V380 software (LaVision BioTec). Emission was detected with HQ535/50-nm and ET605/70-nm filters. A tuneable broad-band Ti:Sa laser (Chameleon, Coherent) was used at 760 nm to capture Alexa Fluor 488 and rhodamine dextran fluorescence. ImageJ software (NIH) was used to generate movies.

### Transmission electron microscopy of bone marrow MKs

For transmission electron microscopy of MKs, bone marrow was flushed from 12- to 16-week-old male mice using Karnovsky fixative (2% PFA, 2.5% glutaraldehyde in 0.1 M cacodylate buffer) and incubated overnight at 4 °C. Subsequently, fatty components of the samples were fixed with 2% osmium tetroxide in 50 mM sodium cacodylate (pH 7.2), stained with 0.5% aqueous uranyl acetate, dehydrated with a graded ethanol series and embedded in Epon 812. Ultrathin sections were stained with 2% uranyl acetate (in 100% ethanol) followed by lead citrate. Images were taken on a EM900 transmission electron microscope (Zeiss).

### Platelet depletion

Circulating platelets were depleted in mice by injection of 20 μg per 30 g body weight anti-GPIbα-antibodies (Emfret, Eibelstadt, Germany) and platelet counts were monitored by flow cytometry for 10 days.

### Immunoblot

Denatured resting or convulxin-stimulated (0.5 μg ml^−1^) platelets or untreated and MgCl_2_ supplemented MKs were lysed and separated by sodium dodecyl sulfate-polyacrylamide gel electrophoresis (SDS–PAGE) and blotted onto polyvinylidene difluoride membranes. NMMIIA (1 μg ml^−1^, #3403, polyclonal, CellSignaling; 1 μg ml^−1^, MP3791; ECM Biosciences), Gapdh (1 μg ml^−1^, G5262, Sigma-Aldrich) or MagT1 (10 μg ml^−1^, AP5056a, Abgent) were probed with the respective antibodies and detected using horseradish peroxidase-conjugated secondary antibodies (0.33 μg ml^−1^) and enhanced chemiluminescence solution (JM-K820-500, MoBiTec). Images were recorded using a MultiImage II FC Light Cabinet (Alpha Innotech cooperation) device. Uncropped immunoblotting images are shown in Supplementary Figs 28 and 29.

### Extraction and sedimentation of the cytoskeleton

Resting or activated (0.1 U ml^−1^ thrombin (10602400001, Roche) platelets (5.7 × 10^7^) or MKs (5 × 10^6^) were lysed in PHEM buffer containing 1% Triton X100, 6 μM taxol (A4667, AppliChem) for microtubule sedimentation or 2 μM phalloidin (A1488, AppliChem) for F/G-actin sedimentation and protease inhibitors (P8340, Sigma-Aldrich). Polymerized and soluble fractions were separated by centrifugation for 30 min at 100.000 g (microtubules) or 16.000 g (F/G-actin), respectively, and 37 °C in a TLA-100 rotor (Beckman Coulter). Total platelet lysates, soluble supernatants (S) and insoluble pellets (P) were supplemented with SDS–PAGE buffer containing 5% β-mercaptoethanol (M6250, Sigma-Aldrich). Samples were separated by SDS–PAGE, blotted onto polyvinylidene difluoride membranes and probed with anti-Tyr-tubulin (0.5 μg ml^−1^, MAB1864 (YL1/2), Milipore), anti-acetylated tubulin (1 μg ml^−1^, sc-23950 (6-11B-1), Santa Cruz Biotechnology Inc.), anti-detyrosinated-tubulin (1 μg ml^−1^, AB3201, Milipore), NMMIIA (1 μg ml^−1^, #3403, polyclonal, CellSignaling; 1 μg ml^−1^, MP3791; ECM Biosciences) or β-actin (1 μg ml^−1^, SAB5500001 (SP124), Milipore) antibodies.

### Cold-induced microtubule disassembly

Microtubules were depolymerized by incubation of platelets at 4 °C; reassembly was allowed by subsequent rewarming at 37 °C. Samples maintained at 4 °C and 37 °C as well as rewarmed platelets were fixed and permeabilized in PHEM buffer supplemented with 4% PFA and 1% IGEPAL CA-630 and allowed to adhere to a PLL-coated coverslips. Samples were immunostained using an anti-β_1_-tubulin (2.5 μg ml^−1^, T4026, clone TUB 2.1, Sigma-Aldrich) or anti-α-tubulin Alexa F488 antibody (3.33 μg ml^−1^, 322588 (B-5-1-2), Invitrogen) and visualized using a Leica TCS SP5 confocal microscope (Leica Microsystems).

### Actin polymerization

Washed platelets were incubated with a DyLight-649–labelled anti-GPIX antibody derivative (20 μg ml^−1)^. Subsequently, platelets were either left unstimulated or were stimulated with the indicated agonists for 2 min. Platelets were fixed with 0.55 volume of 10% paraformaldehyde in PHEM buffer and treated with 0.1 volume 1% Triton X100. Subsequently, platelets were stained with 10 μM phalloidin-fluorescein isothiocyanate (P5282, Sigma-Aldrich) for 30 min and analysed on a FACSCalibur.

### Recruitment of patients

The index case and pedigree members were enrolled to the French ‘Network on the inherited diseases of platelet function and platelet production' (INSERM RBM 01–14). Further subjects with bleeding and platelet disorders (BPD) and with unrelated rare disorders (control data) were enrolled to the BRIDGE-BPD project of the NIHR BioResource—Rare Diseases study (UK REC 13/EE/0325). Here, gene variants were retained in an analysis set if they occur at a frequency of <1:10,000 in the exome aggregation consortium database based on over 60,000 individuals (http://exac.broadinstitute.org/). All cases provided informed written consent at enrolment.

### The BRIDGE bleeding and platelet disorders collection

We searched for cases with coding variants of extreme low frequency in the TPRM7 gene by analysing data from 702 index cases with BPDs of unknown genetic basis recruited to the BRIDGE study of the NIHR BioResource—Rare Diseases. Gene variants were identified in genome sequencing data by comparison with reference human genome build GRCh37 and consequences were predicted using the Ensembl version 75 TPRM7 transcript ENST00000313478. Variants predicted to alter the amino acid sequence of the protein were retained in the analysis set if they occurred at frequencies of <1 in 10,000 out of 67,000 individuals of the Exome Aggregation Consortium (ExAC) database, <1 in 1,000 in the 10,000 subjects of UK10K database and <1 in 100 in 3,453 subjects with unrelated rare disorders or unaffected pedigree members recruited to other branches of the NIHR BioResource. We selected cases with extreme low-frequency variants and on the basis of clinical and laboratory characteristics, which were coded using Human Phenotype Ontology (HPO) terms^50^ retrieved from the BRIDGE-BPD study database. We then focused our further exploration on the cases with extremely rare variants that were unobserved in the nearly 81,000 control DNA samples. The first step was to contact the families to explain the necessity to control these results by performing co-segregation studies. One family met these requirements and additional family members were examined in Paris at a French Centre for inherited platelet diseases.

### Co-segregation studies

Genomic DNA from peripheral blood mononuclear cells was extracted using a commercial DNA purification kit (Qiagen) and exon 17 of TRPM7 was amplified by polymerase chain reaction using the respective primer pairs: TRPM7_Ex17_forw: 5′-ggagaatgtgctctggattc-3′ and TRPM7_Ex17_rev: 5′-gccaatcatccatcttgctc-3′, expected product size 606 bp. PCR products were purified with the help of QIAquick PCR purification kit (Qiagen) and processed for Sanger sequencing.

### Platelet function testing

Platelet function testing of human blood was performed by light transmission turbidometric aggregometry. To this end, PRP (2.5 × 10^5^ platelet per μl) was isolated and stimulated with different concentrations of agonists (ADP, 5 and 10 mM; collagen I, 1 and 5 mg ml^−1^; epinephrine, 5 mM; arachidonic acid, 1 mM; ristocetin, 0.6 and 1.5 mg ml^−1^; and TRAP (thrombin receptor activating peptide), 20 mM). Light transmission was followed over time with PPP set as 100% light transmission.

### Transient expression of WT and p.C721G TRPM7 variants

The TRPM7 p.C721G and p.R902C variants were generated by site-directed mutagenesis on the pcDNA3.1-TRPM7 expression construct^51^ using the QuickChange II XL site-directed mutagenesis kit (200517, Agilent Technologies) with the following primer pairs: Trpm7_C721G_for: 5′-ctggagtaattcaaccggcctcaagttagcgtttc-3′ and Trpm7_C721G_rev: 5′-gaaactgctaacttgaggccggttgaattactccag-3′. The mutation was confirmed by sequencing. For transient expression of WT TRPM7 and the p.C721G variant, human embryonic kidney (HEK) 293 cells were maintained at 37 °C and 5% CO_2_ in Earle's minimal essential medium supplemented with 10% foetal bovine serum, 100 μg ml^−1^ streptomycin and 100 U ml^−1^ penicillin (Invitrogen). Cells were transiently co-transfected with eukaryotic expression vectors encoding WT TRPM7 or the p.C721G and p.R902C variant with 100 ng of an enhanced green fluorescent protein (EGFP) reporter construct (pcDNA3.1) using Lipofectamine2000 (Invitrogen) according to the manufacturer's instructions and processed for patch clamp measurements or confocal microscopy.

### Electrophysiology

Patch clamp experiments were performed at a whole-cell configuration. Currents were elicited by a ramp protocol from −100 to +100 mV over 50 ms acquired at 0.5 Hz and a holding potential of 0 mV. Inward currents were extracted at −80 mV, outward currents at +80 mV and plotted versus time. Data were normalized to cell size as pA pF^−1^. Capacitance was measured using the automated capacitance cancellation function of the EPC10 (HEKA). Nominally Mg^2+^-free extracellular solution contained 140 mM NaCl, 3 mM CaCl_2_, 2.8 mM KCl, 0 mM MgCl_2_, 10 mM HEPES-NaOH, 11 mM glucose (pH 7.2, 300 mOsm). Intracellular solution contained 120 mM Cs-glutamate, 8 mM NaCl, 1 mM MgCl_2_, 10 mM HEPES, 10 mM BAPTA, 5 mM EDTA (pH 7.2, 300 mOsm).

### Image analysis

All images of an experiment were acquired with the same laser power, scan speed, detector settings, processed equally and analysed blinded. Microtubule surface area was determined with the help of Fiji^52^ by thresholding the α-tubulin staining of platelets and MKs using the same settings for both groups. NMMIIA distribution in resting platelets was analysed on three-dimensional surface plots and profile plots with the help of Fiji^52^. Means of the first and last maxima and the mean between the first and last minima of the histograms were determined and the NMMIIA_max_ to NMMIIA_min_ was calculated and depicted. NMMIIA distribution in spread MKs was analysed by grouping and counting the different distribution patterns. The surface of spread platelets was measured by thresholding the F-actin staining. To analyse the distribution/content of NMMIIA in spread platelets the area of the NMMIIA staining was correlated to the total cell size (F-actin) staining.

### Data analysis

The presented results are mean±s.d. from at least three independent experiments per group, if not otherwise stated. Data distribution was analysed using the Shapiro–Wilk test and differences between control and knockout mice were statistically analysed using Student's *t*-test or Wilcoxon–Mann–Whitney test, respectively. *P*-values<0.05 were considered as statistically significant *P<0.05; **P<0.01; ***P<0.001. Results with a *P* value>0.05 were considered as not significant (NS).

## Additional information

**How to cite this article:** Stritt, S. *et al*. Defects in TRPM7 channel function deregulate thrombopoiesis through altered cellular Mg^2+^ homeostasis and cytoskeletal architecture. *Nat. Commun.* 7:11097 doi: 10.1038/ncomms11097 (2016).

## Supplementary Material

Supplementary Information Supplementary Figures 1-29, Supplementary Tables 1-3 and Supplementary Note

Supplementary Movie 1 Representative intravital two-photon microscopy *time-lapse* movie of bone marrow MKs in the skull of a control mouse, showing proplatelet formation. Scale bar, 50 μm.

Supplementary Movie 2 Representative intravital two-photon microscopy *time-lapse* movie of bone marrow MKs in the skull of a Trpm^7fl/fl-Pf4Cre^ mouse with mature MKs attempting to form proplatelets but fail to do so. Scale bar, 50 μm.

Supplementary Movie 3 Representative intravital two-photon microscopy *time-lapse* movie of bone marrow MKs in the skull of a Trpm^7fl/fl-Pf4Cre^ mouse during proplatelet formation (arrow) showing short and bulky proplatelet protrusion that remains attached to the MK body. Scale bar, 50 μm.
