# Supplementary material for: Defects in TRPM7 channel function deregulate thrombopoiesis through altered cellular Mg2+ homeostasis and cytoskeletal architecture
Source: Nat Commun. 2016 Mar 29;7:11097. doi: 10.1038/ncomms11097 (PMC4820538; doi:10.1038/ncomms11097)
Supplement: Supplementary Information — Supplementary Figures 1-29, Supplementary Tables 1-3 and Supplementary Note [file ncomms11097-s1.pdf]

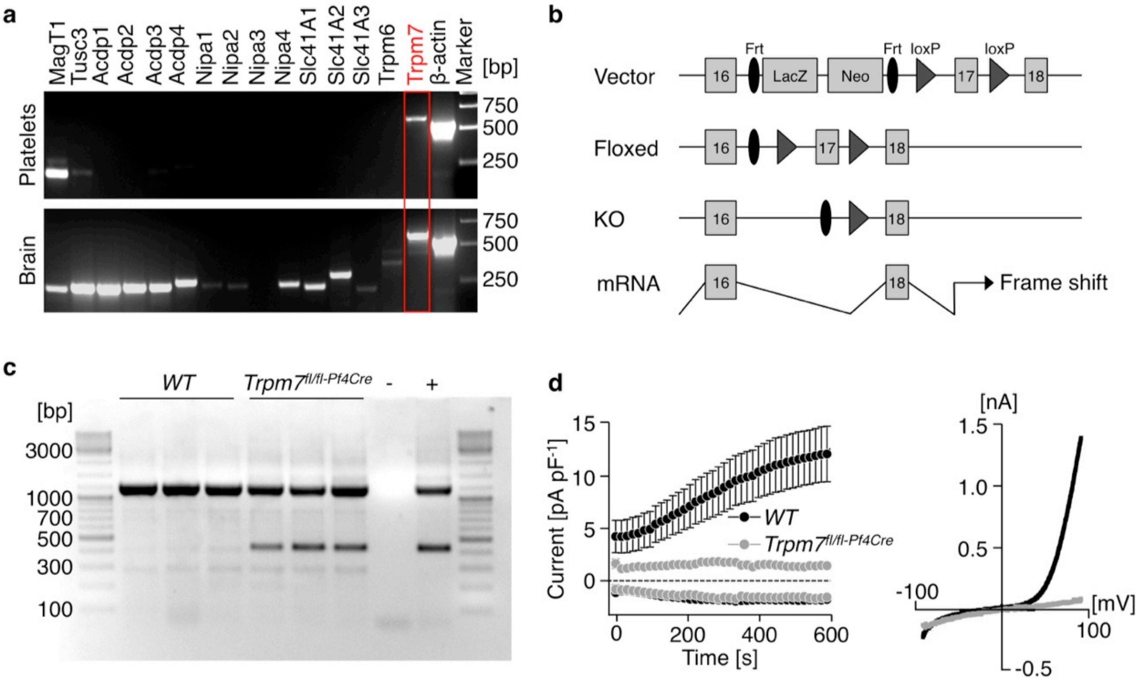

Supplementary Figure 1. *Trpm7* is prominently expressed in mouse platelets. (**a**) Characterisation of Mg^{2+} transporter/channel expression in mouse platelets using reverse transcriptase PCR. (**b**) Targeting strategy of *Trpm7*$^{fl/fl\text{-}Pf4Cre}$ mice leading to a non-functional TRPM7 channel protein. (**c**) Genotyping of *Trpm7*$^{fl/fl\text{-}Pf4Cre}$ mice via PCR reveals successful recombination of the two loxP sites, yielding a 1200 bp product for the floxed (*WT*) and a 400 bp fragment for the recombined allele. (**d**) Efficiency of the targeting strategy was revealed by whole cell patch clamp measurements on bone marrow-derived megakaryocytes from *WT* and *Trpm7*$^{fl/fl\text{-}Pf4Cre}$ mice. Measurements have been conducted in absence of extracellular Mg^{2+} to enhance current sizes. Currents were elicited by a ramp protocol from -100 to +100 mV over 50 ms acquired at 0.5 Hz. Left panel: Inward current amplitudes were extracted at -80 mV, outward currents at +80 mV and plotted versus time of the experiment. Values are normalised to cell size as pA pF^{-1} and represent mean ± sem. The depletion of intracellular Mg^{2+} leads to the development of characteristic TRPM7-like currents in *WT* MKs (black circles, n = 13), whereas TRPM7 currents were abolished in *Trpm7*$^{fl/fl\text{-}Pf4Cre}$ MKs (red circles, n = 10). Right panel: Representative current-voltage relationships extracted at 600 s. *WT* cells show an I V^{-1}-relationship characteristic for TRPM7 (black trace), which are absent in *Trpm7*$^{fl/fl\text{-}Pf4Cre}$ MKs (light grey trace).

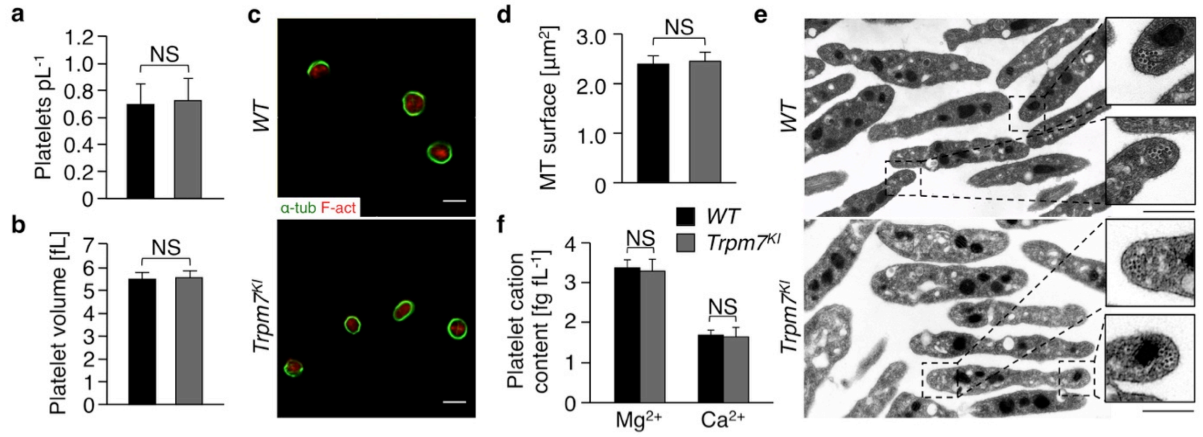

Supplementary Figure 2. Abolished Trpm7 α-kinase activity does not cause macrothrombocytopenia in mice. (**a, b**) Trpm7 kinase-dead (*Trpm7*^{KI}) mice display a normal platelet count (**a**) and size (**b**) as determined with an automated blood cell analyser (Sysmex KX-21N™). Values are mean ± s.d. (n = 13). (**c - e**) Confocal (**c**) and transmission electron microscopy (**e**) reveal normal platelet morphology and organisation of microtubules (**d**) into the marginal band (**e**, inlays). Scale bars, 3 μm (for confocal images). Scale bars, 1 μm (for TEM images). All images are representative of at least 5 animals. Values in **d** are mean ± s.d. (n = 5; 200 platelets). (**f**) Total platelet cation content was determined by inductively coupled plasma mass spectrometry. Values are mean ± s.d. (n = 5). Unpaired Student's t-test; NS, non-significant. All depicted panels are representative of at least 3 independent experiments.

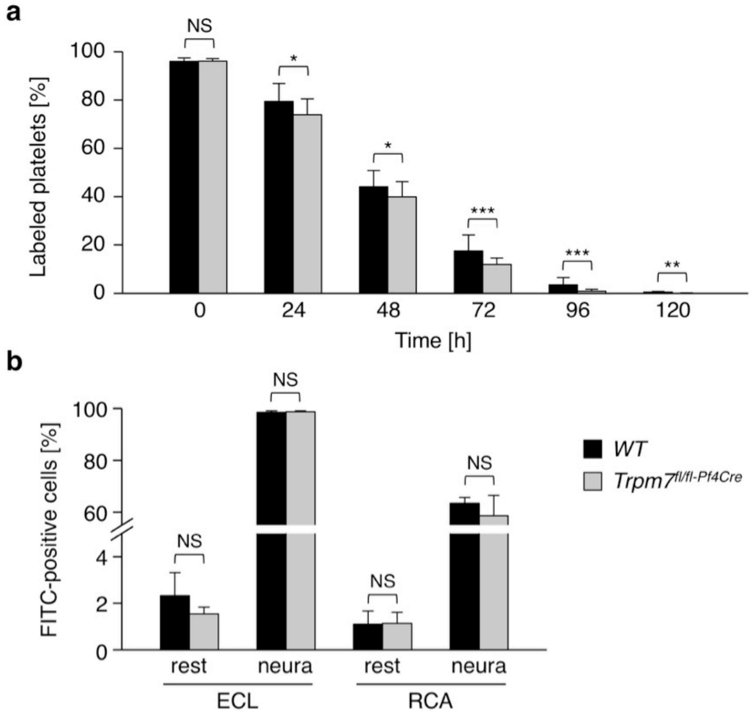

Supplementary Figure 3. Mildly accelerated clearance of *Trpm7*$^{fl/fl-Pf4Cre}$ platelets. (**a**) Platelet life span was measured by injection of a DyLight 488-conjugated anti-GPIX antibody. (**b**) Platelet terminal galactose levels (desialylation) were measured by binding of FITC-conjugated *Erythrina cristagalli* lectin (ECL) and *Ricinus communis* agglutinin (RCA). Neuraminidase treatment (2.5 mU of α2–3,6,8 neuraminidase for 15 minutes at 37°C served as positive control. Values are mean ± s.d. (n = 5; 3 independent experiments). Unpaired Student's t-test; ***P<0.001; **P<0.01; *P<0.05; NS, non-significant

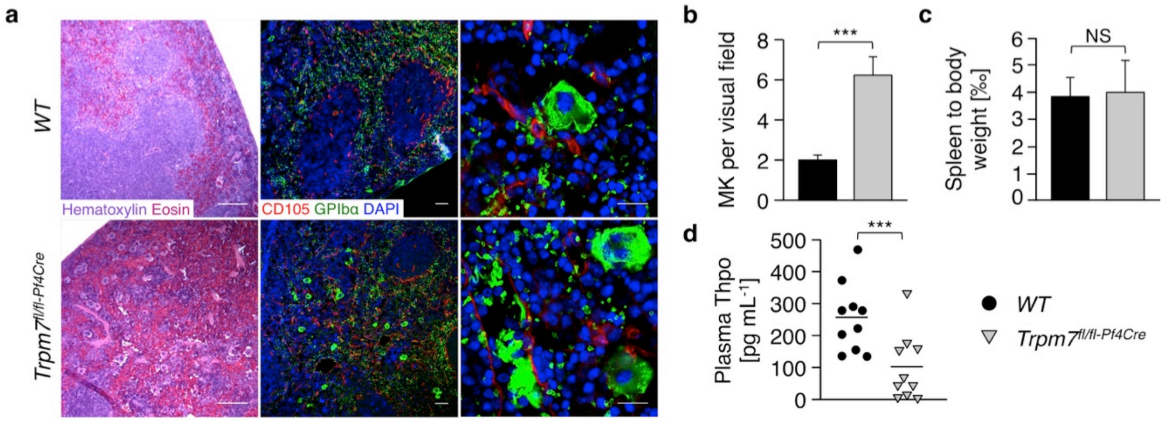

Supplementary Figure 4. Aberrant architecture of the red and white pulp in spleens of *Trpm7*$^{fl/fl\text{-}Pf4Cre}$ mice. (**a**) Hematoxylin-eosin and immunostaining on spleen sections reveals an expansion of the red pulp. Scale bars, 50 μm (in the left and mid panel). Scale bars, 15 μm (in the right panel). (**b**) Quantification of splenic MKs per visual field (328 x 246 μm). Values are mean ± s.d. (n = 6; 20 visual fields were analysed for each measurement). (**c**) Normal ratio of spleen to body weight in *Trpm7*$^{fl/fl\text{-}Pf4Cre}$ mice. Values are mean ± s.d. (n = 6). (**d**) In line with the increased number of MKs, plasma thrombopoietin (Thpo) levels were decreased in *Trpm7*$^{fl/fl\text{-}Pf4Cre}$ mice. Each symbol represents one mouse (n = 10). Horizontal lines represent mean. All images are representative of at least 5 animals and 3 independent experiments. Unpaired Student's t-test; ****P*<0.001; NS, non-significant.

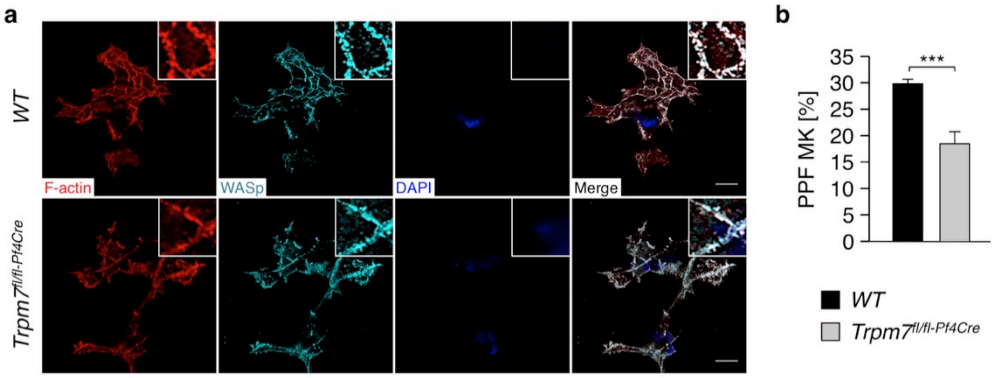

Supplementary Figure 5. *Trpm7$^{fl/fl-Pf4Cre}$* bone marrow-derived MKs show unaltered podosome formation. (**a**) Confocal images of bone marrow-derived MKs (n = 7) spread (3 h) on collagen I and stained for F-actin (red), DAPI (blue) and WASp (cyan) serving as a marker for podosomes. Podosomes are highlighted in inlays. Scale bars, 25 μm. (**b**) Proplatelet formation of bone marrow-derived MKs. Values are mean ± s.d. (n = 5; 3 independent experiments). Unpaired Student's t-test; ****P*<0.001.

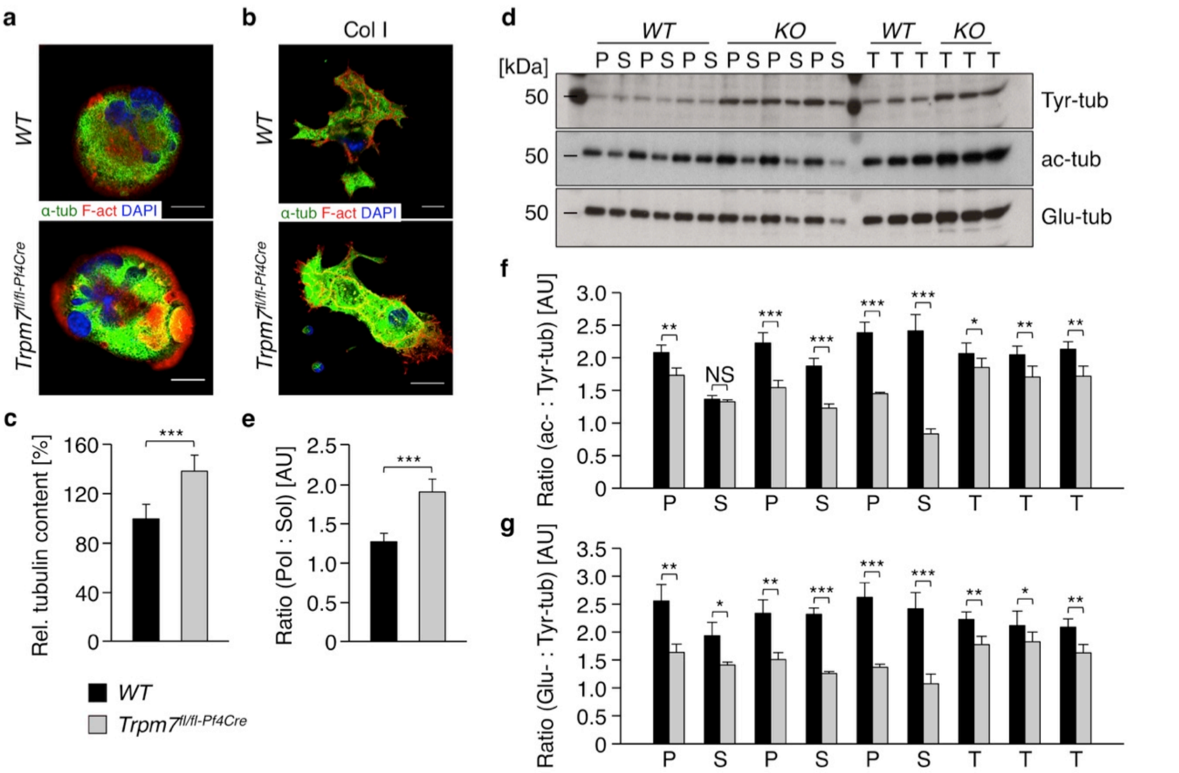

Supplementary Figure 6. Aberrant cytoskeletal organisation of bone marrow-derived *Trpm7*$^{fl/fl\text{-}Pf4Cre}$ MKs. (**a, b**) Confocal images of resting (**a**) and on collagen I (50 μg μL^{-1}) spread (**b**) bone marrow-derived MKs stained for F-actin (red), α-tubulin (green) and DAPI (blue). Scale bars, 25 μm. All images are representative of at least 5 animals. (**c**) Quantification of integrated fluorescence intensity of the α-tubulin staining revealed an increased content of tubulin in resting (**a**) and spread (**b**) *Trpm7*$^{fl/fl\text{-}Pf4Cre}$ MKs. Values are mean ± s.d. (n = 6; 200 MKs). (**d**) The tubulin cytoskeleton of resting bone marrow-derived MKs was isolated by ultracentrifugation and immunoblotted to detect dynamic Tyr-tubulin and post-translational modifications of stable microtubules by the analysis of acetylated (ac)- or detyrosinated (Glu)-tubulin. Insoluble fraction (pellet, P); soluble fraction (supernatant, S); T, total protein. (**e - g**) Densitometry revealed an increased prevalence of polymerised microtubules (**e**) in bone marrow-derived Trpm7-deficient MKs that was characterised by a markedly reduced ratio of stable acetylated (**f**) and detyrosinated (**g**) microtubules to highly dynamic tyrosinated microtubules. Values are mean ± s.d. (n = 6). Unpaired Student's t-test; ****P*<0.001; ***P*<0.01; **P*<0.05; NS, not significant. All panels are representative of at least 3 independent experiments.

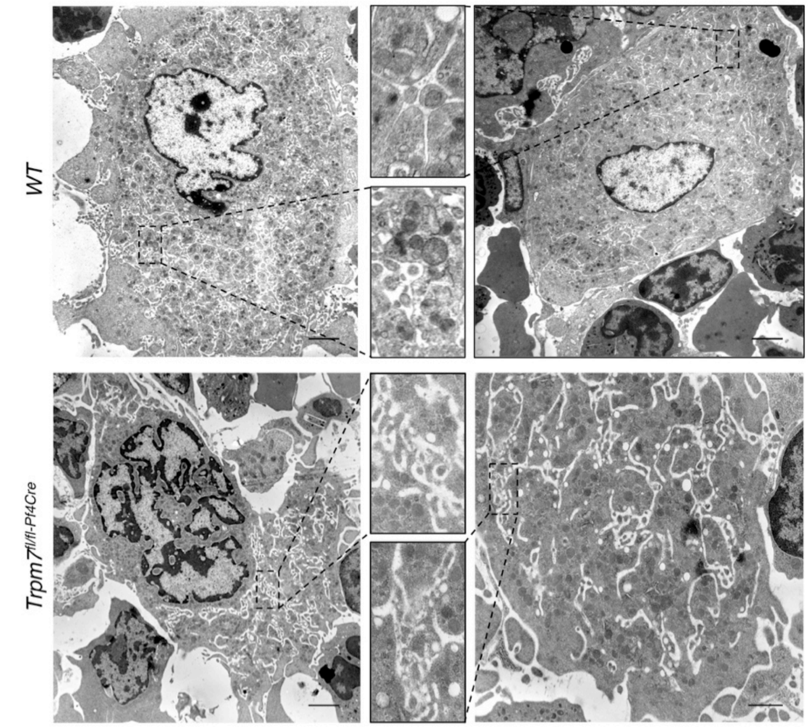

Supplementary Figure 7. Mature *WT* and *Trpm7*$^{fl/fl\text{-}Pf4Cre}$ bone marrow MKs. In *Trpm7*$^{fl/fl\text{-}Pf4Cre}$ MKs tortuous membrane complexes were observed (inlays). Scale bars, 2.5 μm. Images are representative of at least 5 animals and 3 independent experiments.

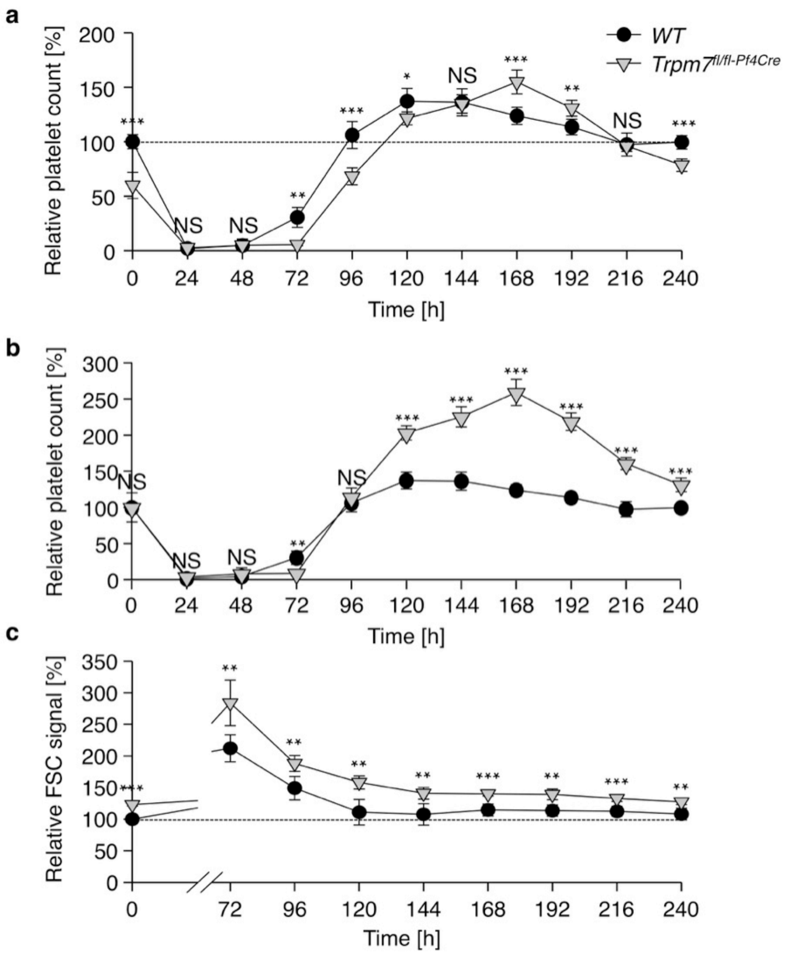

Supplementary Figure 8. Impaired platelet production following antibody-induced thrombocytopenia in *Trpm7*$^{fl/fl\text{-}Pf4Cre}$ mice. Platelet (**a, b**) count and (**c**) size in control and *Trpm7*$^{fl/fl\text{-}Pf4Cre}$ mice were monitored for 10 days after platelet depletion with an anti-GPIbα antibody (Emfret). Symbols are mean ± s.d. (n = 5; representative of 3 independent experiments). Unpaired Student's t-test; ****P*<0.001; ***P*<0.01; **P*<0.05; NS, non-significant.

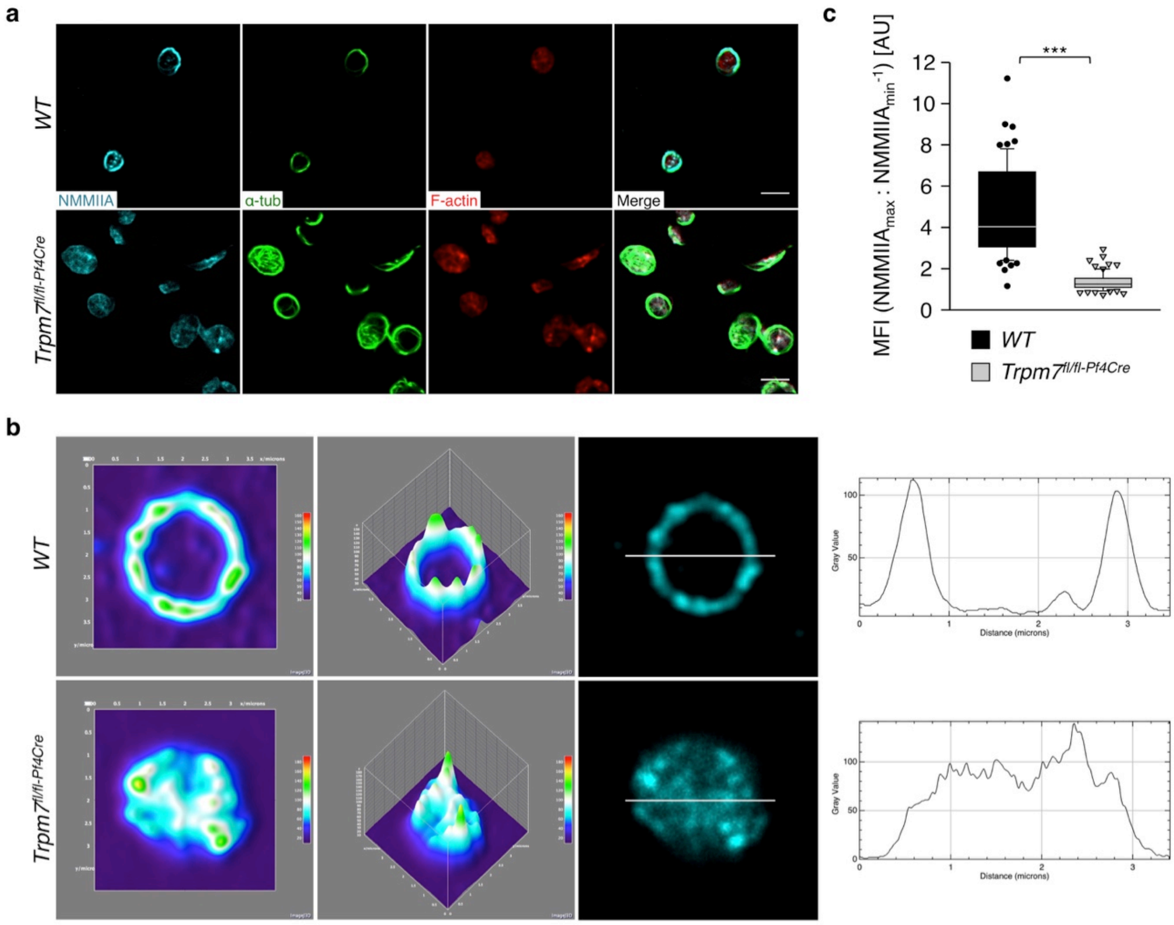

Supplementary Figure 9. Altered localisation of NMMIIA in platelets from *$Trpm7^{fl/fl-Pf4Cre}$* mice. (**a**) Poly-L-lysine-immobilised resting platelets were permeabilised and stained for F-actin (red) α-tubulin (green), NMMIIA (cyan) and analysed by confocal microscopy. Scale bars, 3 μm. (**b**) 3D surface plots (left panels) and profile plots (right panels) of NMMIIA staining were prepared with the help of Fiji[52]. (**c**) Image analysis (ratio of the mean of the first and last maxima and the mean between the first and last minima) revealed an aberrant distribution of NMMIIA in platelets from *$Trpm7^{fl/fl-Pf4Cre}$* mice as compared with *WT* controls. Box plots display first and third quartiles and whiskers mark minimum and maximum values unless exceeding 1.5x IQR of at least 70 platelets; symbols represent outliers and the horizontal line displays median. Wilcoxon-Mann-Whitney-test; ***P<0.001.

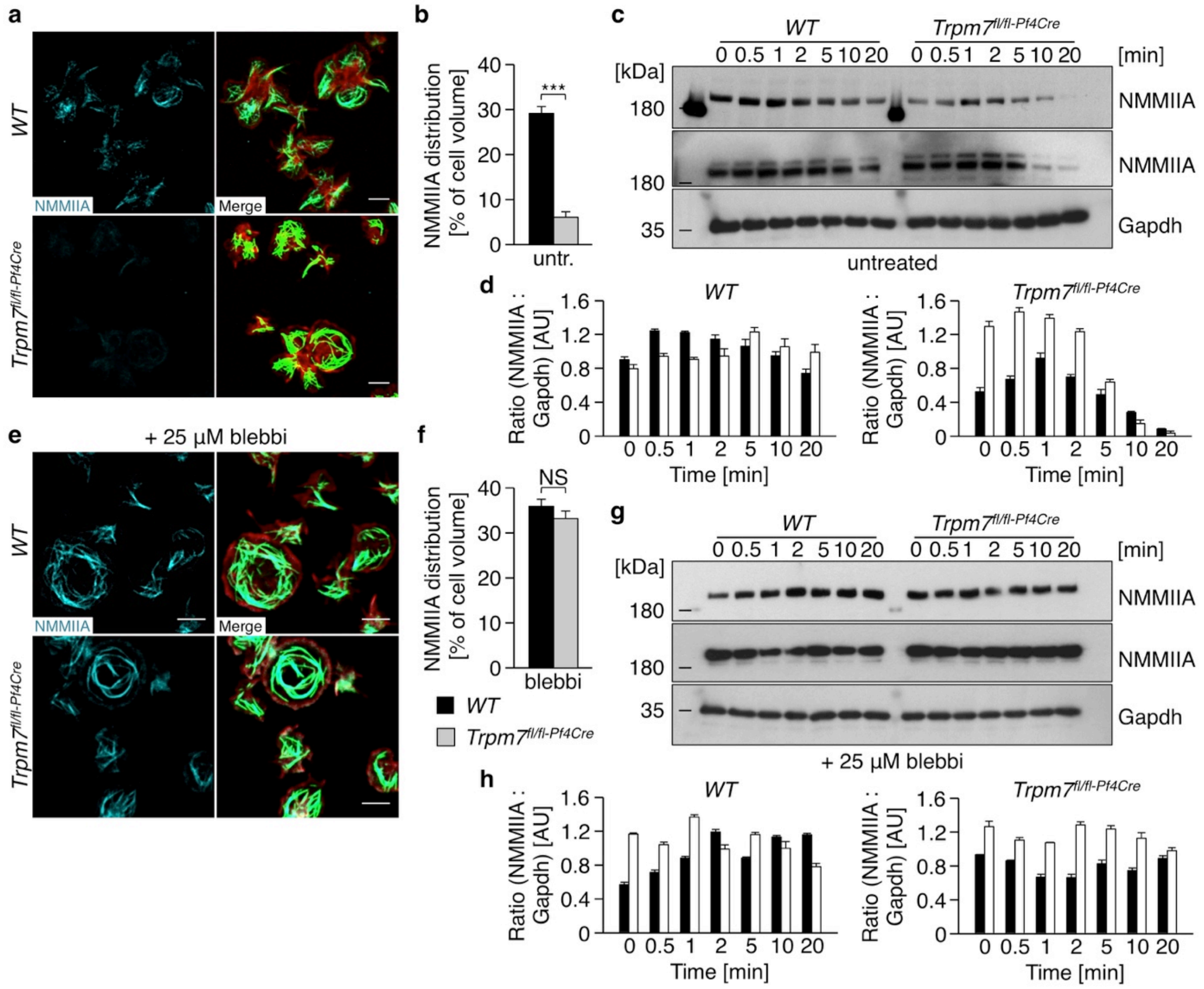

Supplementary Figure 10. Blebbistatin prevents degradation of NMMIIA in *Trpm7*$^{fl/fl\text{-}Pf4Cre}$ platelets. (**a**) Confocal images of spread control and *Trpm7*$^{fl/fl\text{-}Pf4Cre}$ platelets stained for F-actin (red), α-tubulin (green) and NMMIIA (cyan). Scale bars, 3 *µ*m. (**b**) Quantification of the relative NMMIIA surface coverage per platelet revealed a marked decrease of NMMIIA in *Trpm7*$^{fl/fl\text{-}Pf4Cre}$ platelets. (**c, d**) Untreated platelets were stimulated with 0.5 *µ*g mL^{-1} convulxin (Cvx), lysed after the indicated time points and processed for immunoblotting (**c**). Detection of NMMIIA (230 kDa) was performed with two anti-NMMIIA antibodies. Gapdh (36 kDa) served as loading control. Densitometric analyses (**d**) revealed a rapid degradation of NMMIIA in *Trpm7*$^{fl/fl\text{-}Pf4Cre}$ but not in *WT* platelets. (**e - h**) Pretreatment of *Trpm7*$^{fl/fl\text{-}Pf4Cre}$ platelets with 25 *µ*M blebbistatin prevented the spreading- (**e, f**) or Cvx stimulation-induced (**g, h**) degradation of NMMIIA. Scale bars, 3 *µ*m. All images are representative of at least 5 animals and 3 independent experiments. Values are mean ± s.d. (n = 6; 200 platelets). Unpaired Student's t-test; ****P*<0.001; NS, not significant.

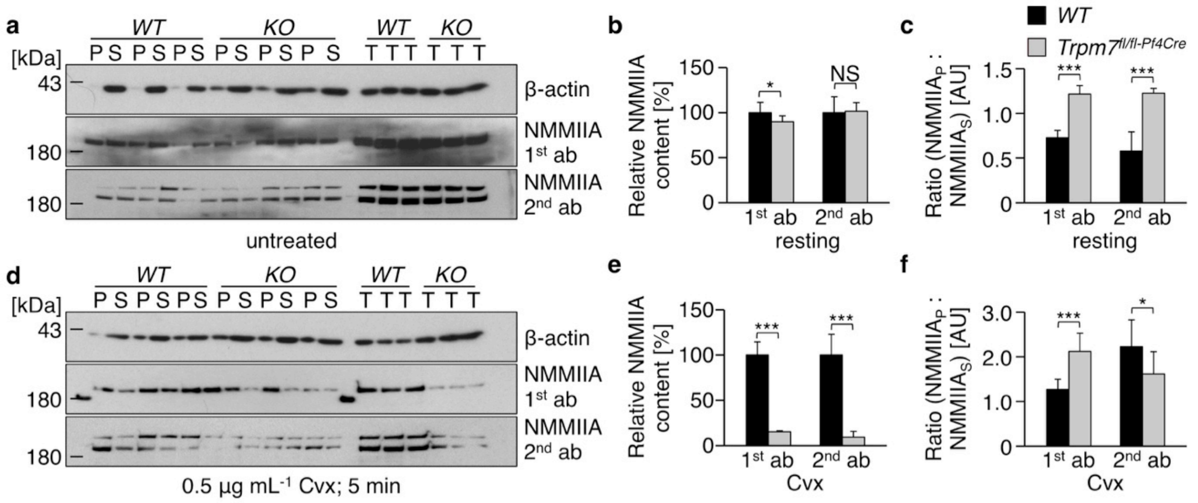

Supplementary Figure 11. Increased NMMIIA activity and rapid degradation in *Trpm7*$^{fl/fl-Pf4Cre}$ platelets. (**a** - **c**) Cortical F-actin meshwork of resting platelets was isolated by ultracentrifugation at 16.000 g and immunoblotted to detect β-actin and NMMIIA using two different antibodies (**a**). Insoluble, cross-linked fraction (pellet, P); soluble fraction (supernatant, S); T, total protein. Densitometry revealed an unaltered NMMIIA content in *Trpm7*$^{fl/fl-Pf4Cre}$ platelets under resting conditions (**b**) with an increased amount of NMMIIA in the pellet fraction (**c**) containing cross-linked F-actin filaments. (**d** - **f**) Stimulation (10 min) of *Trpm7*$^{fl/fl-Pf4Cre}$ but not *WT* platelets with 0.5 μg mL^{-1} Cvx resulted in a pronounced reduction of the total NMMIIA content (**d, e**) and an increased incorporation of NMMIIA into the polymerised (**f**) and cross-linked fraction. All images are representative of at least 6 animals and 3 independent experiments. Values are mean ± s.d. (n = 6). Unpaired Student's t-test; ****P*<0.001; **P*<0.05; NS, not significant.

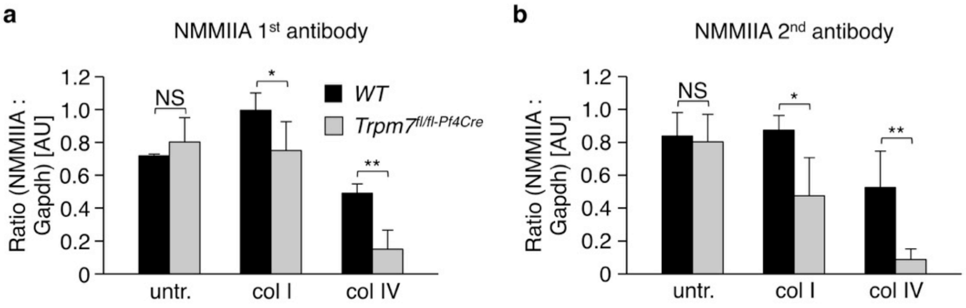

Supplementary Figure 12. Collagen I and IV stimulate degradation of NMMIIA in bone marrow-derived *Trpm7*$^{fl/fl\text{-}Pf4Cre}$ MKs. (**a, b**) Densitometry revealed a decreased content of NMMIIA upon cultivation of bone marrow-derived Trpm7-deficient MKs in the presence of collagen I or IV (each 10 μg cm^{-2}). Values are mean ± s.d. (n = 6; representative of 3 independent experiments). Unpaired Student's t-test; ***P*<0.01; **P*<0.05; NS, not significant.

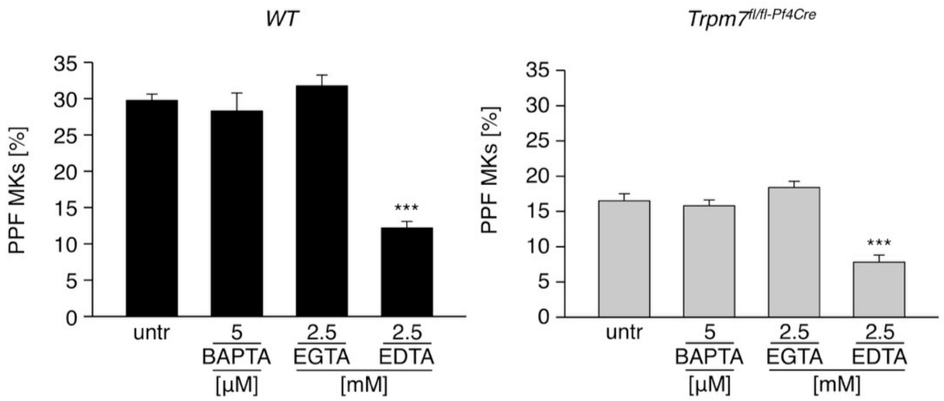

Supplementary Figure 13. Deregulated [Mg^{2+}]$_i$ likely accounts for the impaired proplatelet formation of bone marrow-derived *Trpm7$^{fl/fl\text{-}Pf4Cre}$* MKs. Pretreatment (90 min) of bone marrow-derived MK cultures with the Ca^{2+} chelators BAPTA-AM (1,2-bis(2-aminophenoxy)ethane-N,N,N′,N′-tetraacetic acid tetrakis(acetoxymethyl ester) or EGTA (ethylene glycol-bis(2-aminoethylether)-*N,N,N′,N′*-tetraacetic acid) did not interfere with proplatelet formation, whereas the non-selective cation chelator EDTA (ethylenediaminetetraacetic acid) strongly impaired proplatelet formation in both *WT* and *Trpm7$^{fl/fl\text{-}Pf4Cre}$* bone marrow-derived MKs. Values are mean ± s.d. (n = 5; representative of 3 independent experiments). Unpaired Student's t-test; ***P<0.001.

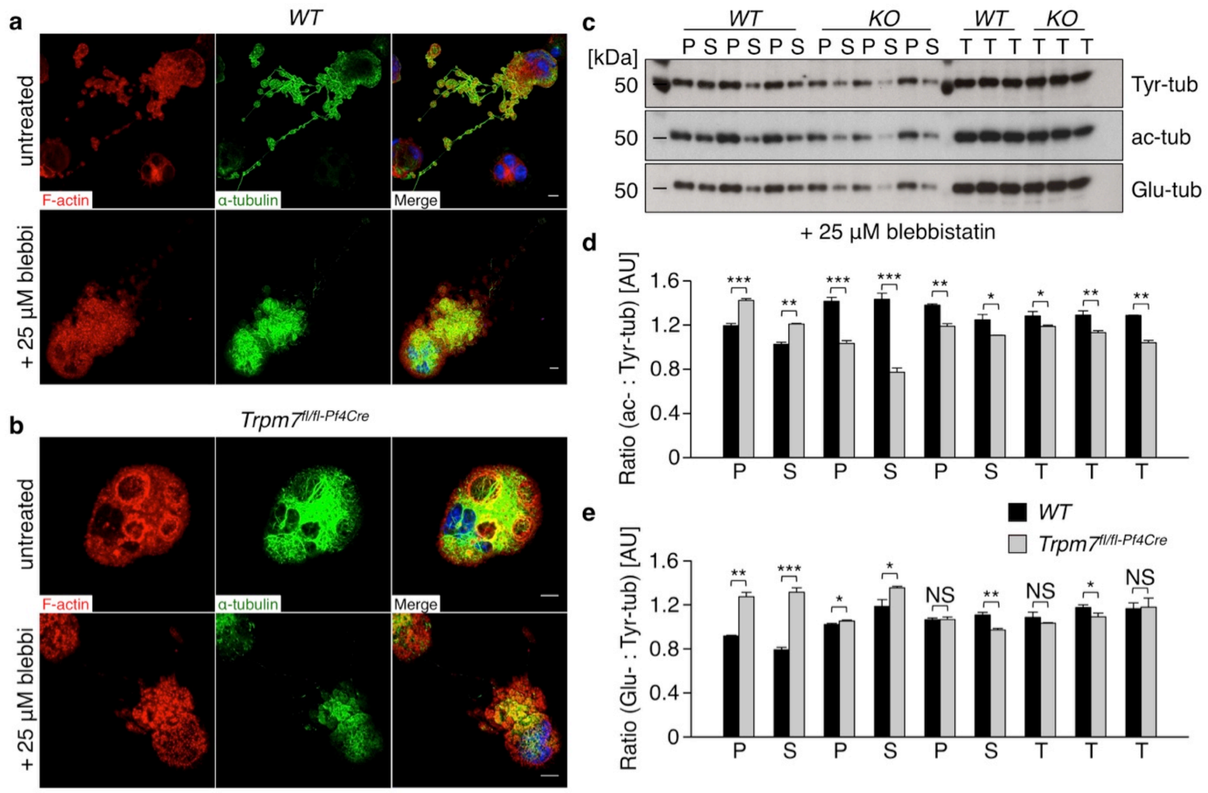

Supplementary Figure 14. Blebbistatin treatment interferes with cytoskeletal organisation of foetal liver- and bone marrow-derived MKs. (**a, b**) Confocal images of untreated or blebbistatin-treated (25 μM) foetal liver-derived MKs from control (**a**) and *Trpm7*$^{fl/fl\text{-}Pf4Cre}$ (**b**) mice stained for F-actin (red), α-tubulin (green) and DAPI (blue). Scale bars, 10 μm. (**c**) Tubulin cytoskeleton of blebbistatin-treated (25 μM) bone marrow-derived MKs was isolated by ultracentrifugation and immunoblotted to detect dynamic tyrosinated (Tyr)-tubulin and post-translational modifications of stable microtubules by the analysis of acetylated (ac)- or detyrosinated (Glu)-tubulin. Insoluble fraction (pellet, P); soluble fraction (supernatant, S); T, total protein. (**d, e**) Densitometry revealed that blebbistatin treatment reduced both the ratio of stable acetylated (**f**) and detyrosinated (**g**) microtubules to highly dynamic tyrosinated microtubules. Values are mean ± s.d. (n = 6). Unpaired Student's t-test; ***P<0.001; **P<0.01; *P<0.05; NS, not significant. All displayed panels are representative of at least 5 animals and 3 independent experiments.

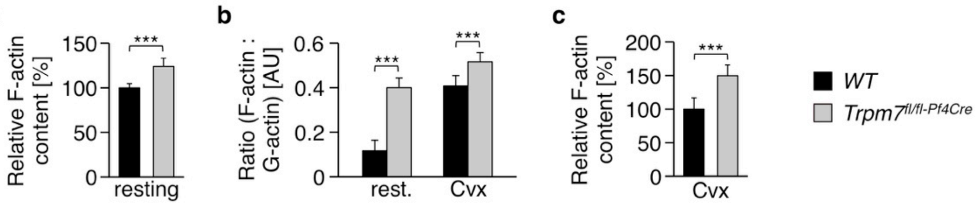

Supplementary Figure 15. Increased F-actin content in *Trpm7$^{fl/fl-Pf4Cre}$* platelets. The total content of β-actin was increased in Trpm7-deficient platelets (**a, c**) and resulted in an increased prevalence of polymerised, cross-linked actin filaments (**b**), particularly under resting conditions as revealed by densitometric analyses. Values are mean ± s.d. (n = 6). Unpaired Student's t-test; ****P*<0.001.

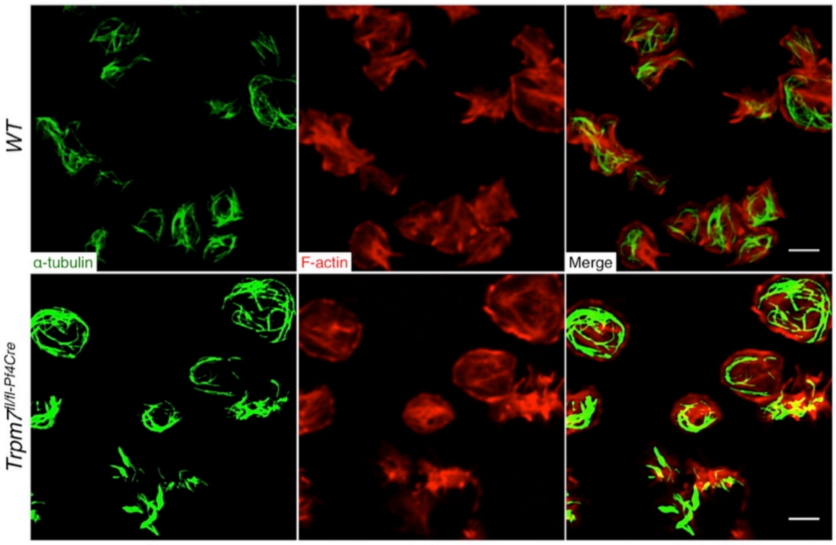

Supplementary Figure 16. Accelerated spreading of *Trpm7*$^{fl/fl\text{-}Pf4Cre}$ platelets on fibrinogen. Confocal images of spread platelets (15 min, 100 μg mL^{-1} fibrinogen) stained for F-actin (red) and α-tubulin (green). Scale bars, 3 μm. All images are representative of at least 5 animals and 3 independent experiments.

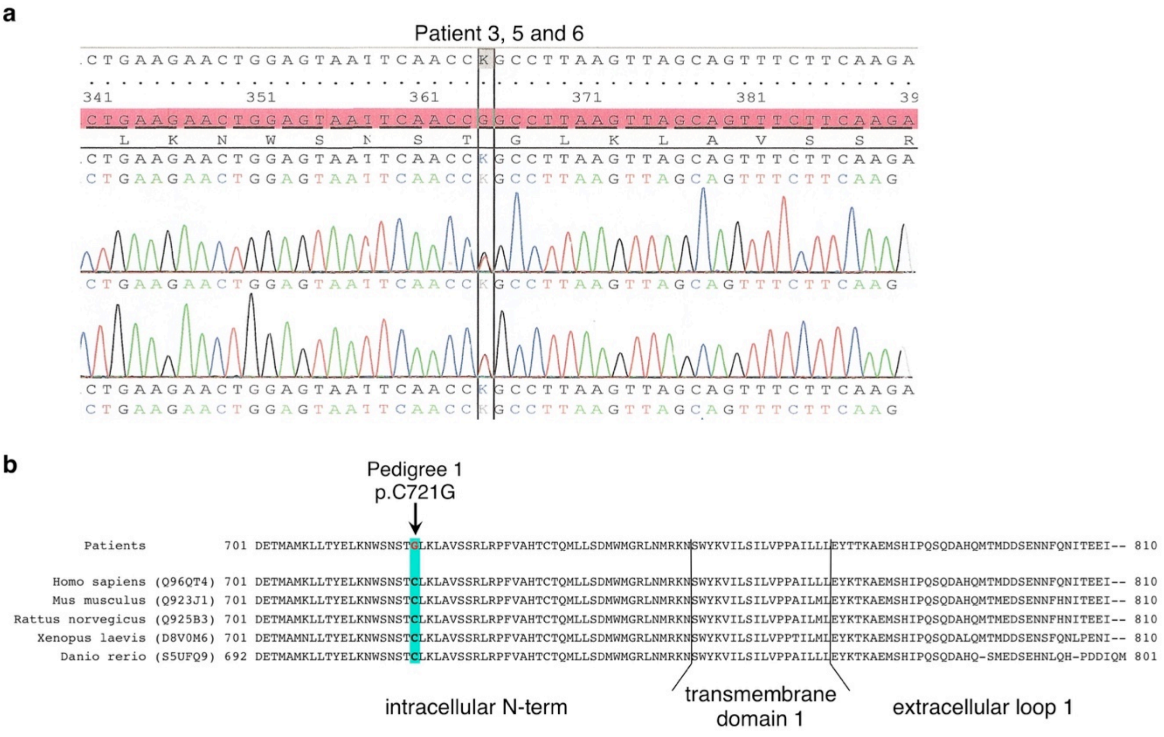

Supplementary Figure 17. The *TRPM7* p.C721G variant co-segregates with macrothrombocytopenia. (**a**) DNA sequencing confirms that patients 3, 5 and 6 carry the p.C721G variant heterozygous. (**b**) Sequence alignment of *TRPM7* coding sequence from different species of different taxa highlights the high sequence conservation.

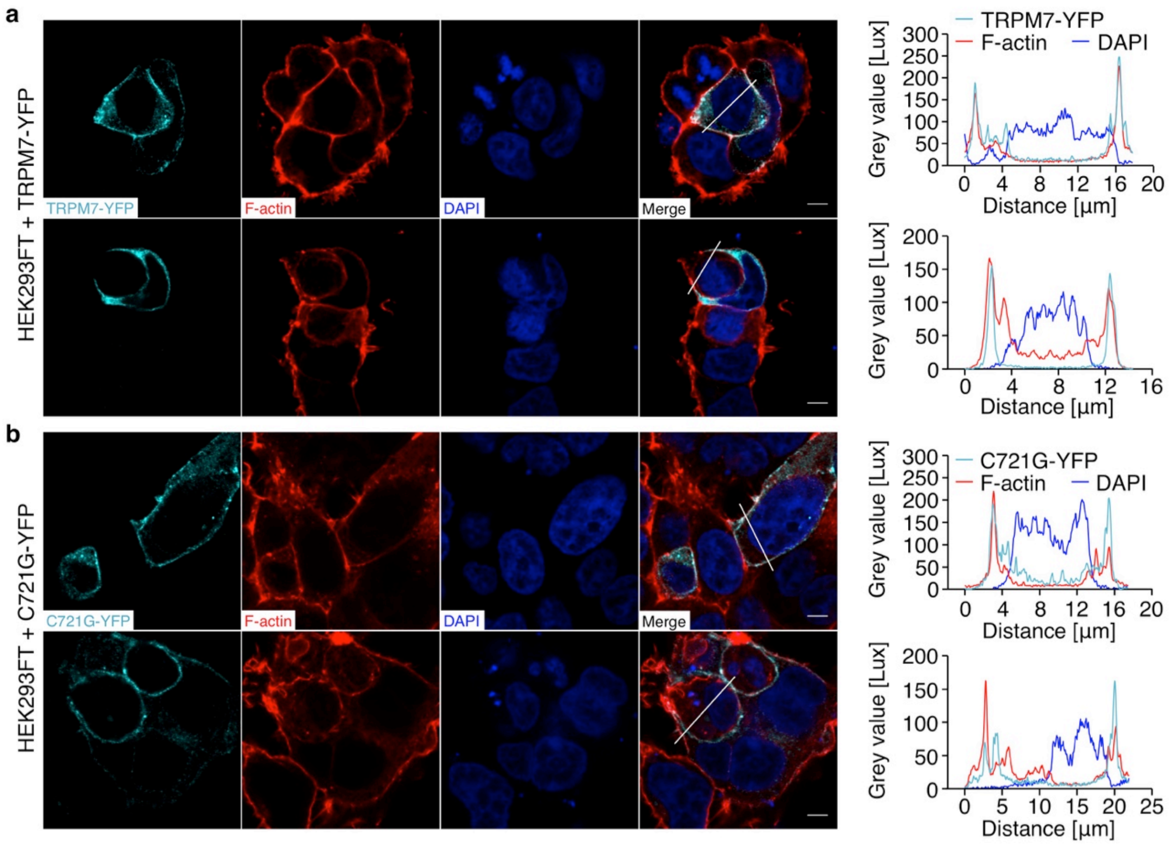

Supplementary Figure 18. The TRPM7 p.C721G-YFP variant localises to the plasma membrane of HEK293FT cells. (**a, b**) HEK293FT cells were grown on cover slips and transfected with *TRPM7*-*WT*-YFP (**a**) or *TRPM7*-p.C721G-YFP (**b**) expression constructs. 48 hours after transfection cells were permeabilised and stained for F-actin (red), DNA (blue) and analysed by confocal microscopy. Scale bars, 5 μm. Profile plots along the white lines in the merged images (right panels) were prepared with the help of Fiji[52] and reveal a localisation of the YFP-tagged *WT* and p.C721G TRPM7 proteins to the actin rich cell cortex. Images are representative of 4 independent experiments.

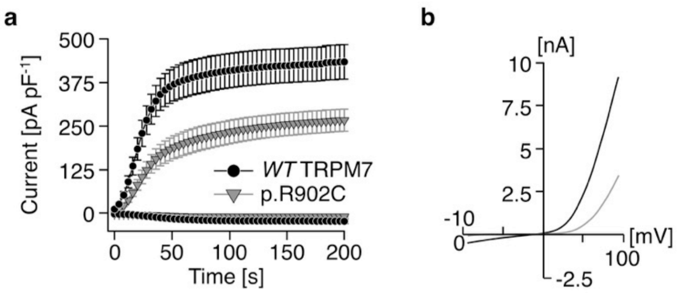

Supplementary Figure 19. The TRPM7 p.R902C variant displays impaired channel activity. Whole currents were measured in mock transfected HEK293 (n = 8), and cells overexpressing *WT* TRPM7 (n = 7) or the p.R902C variant (n = 10) revealed reduced channel activity. Left panel: Inward current amplitudes were extracted at -80 mV, outward currents at +80 mV and plotted versus time of the experiment. Values are normalised to cell size as pA pF^{-1} and represent mean ± sem. The depletion of intracellular Mg^{2+} leads to the development of characteristic TRPM7-like currents in HEK293 cells overexpressing *WT* TRPM7, whereas TRPM7 currents were substantially reduced in p.R902C overexpressing HEK293 cells. Right panel: Representative current-voltage relationships extracted at 200 s. All depicted panels are representative of at least 3 independent experiments.

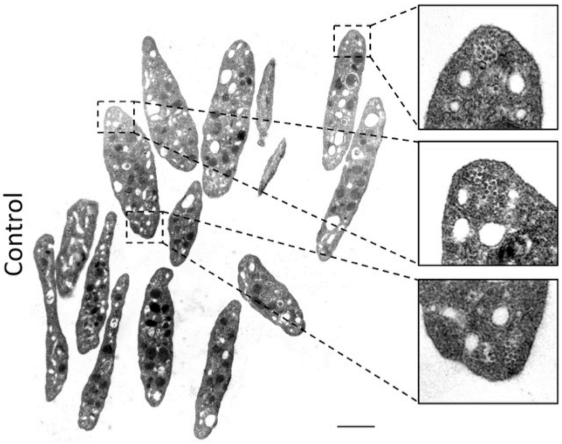

Supplementary Figure 20. TEM image of resting platelets from healthy controls show organisation of microtubules into the characteristic marginal band. Scale bar, 1 μm.

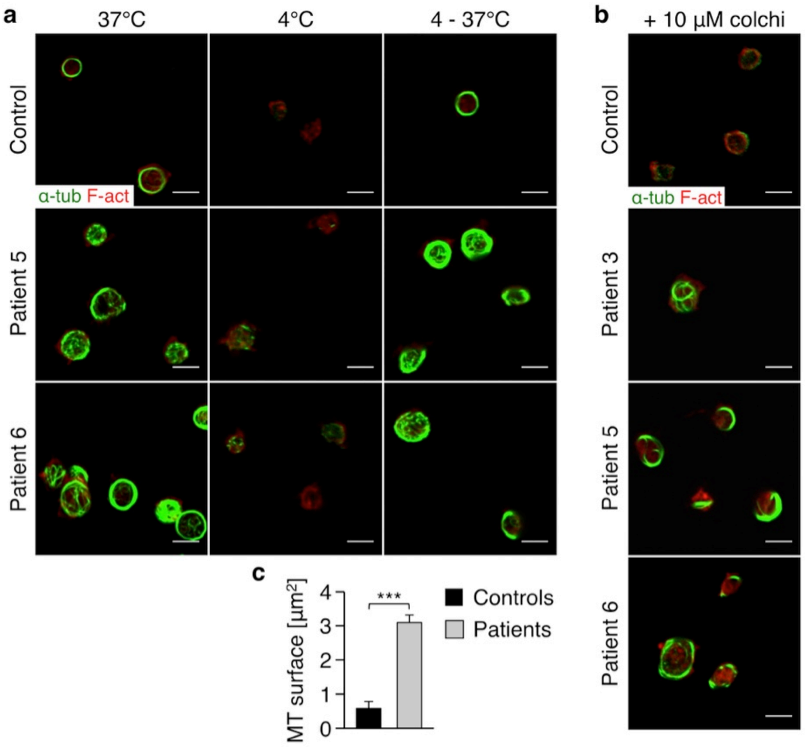

Supplementary Figure 21. Microtubules in platelets from patients with the *TRPM7* p.C721G variant are less prone to colchicine-mediated disassembly. (**a**, **b**) Resting platelets of healthy controls and patients 3, 5 and 6 were subjected to cold-challenge (**a**) or incubated with 10 μM colchicine (**b**), fixed, allowed to adhere to poly-L-lysine-coated cover slips, stained for α-tubulin (green) and F-actin (red) and analysed by confocal microscopy. Scale bars, 3 μm. (**c**) Image analysis revealed an increased prevalence of microtubules upon challenge with colchicine (10 μM) for 30 minutes. Values are mean ± s.d. (n = 3 controls; 200 platelets). Unpaired Student's t-test; ***P<0.001.

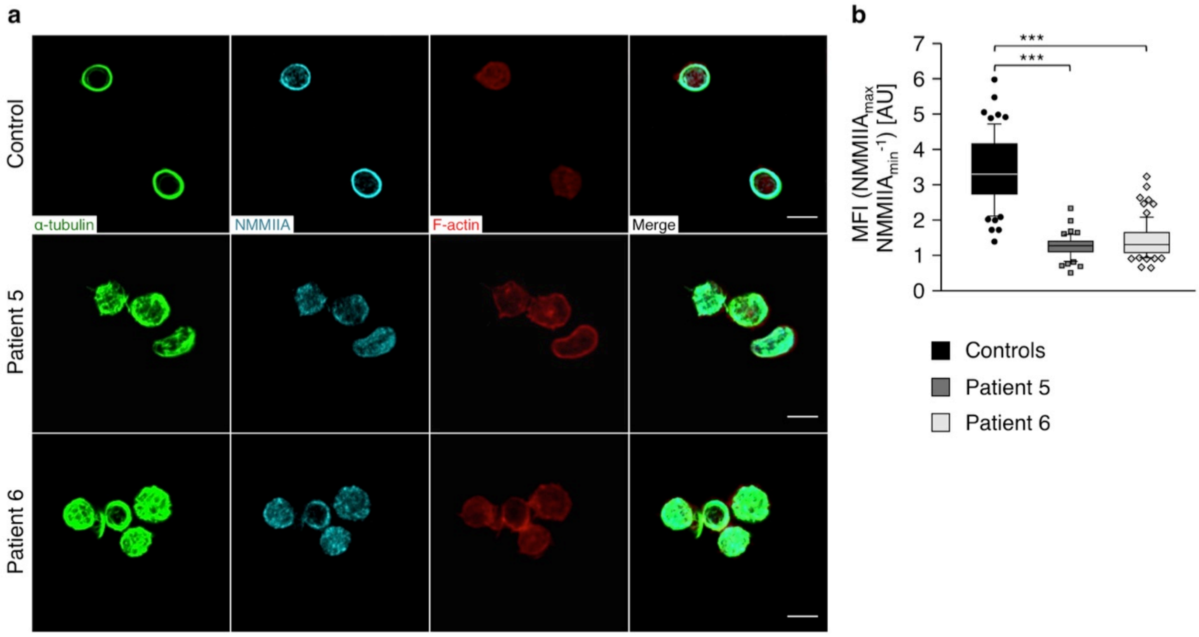

Supplementary Figure 22. Altered localisation of NMMIIA in platelets from patients with the *TRPM7* p.C721G variant. (**a**) Resting platelets of healthy controls, patient 5 and 6 were fixed on poly-L-lysine-coated slides and stained for α-tubulin (green), F-actin (red) and NMMIIA (cyan). Scale bars, 3 μm. (**b**) Image analysis revealed an aberrant distribution of NMMIIA in platelets from patients with variants in *TRPM7* as compared with healthy controls. Box plots display first and third quartiles and whiskers mark minimum and maximum values unless exceeding 1.5x IQR of at least 70 platelets (n = 3 controls); symbols represent outliers and the horizontal line displays median. Wilcoxon-Mann-Whitney-test; ***P<0.001.

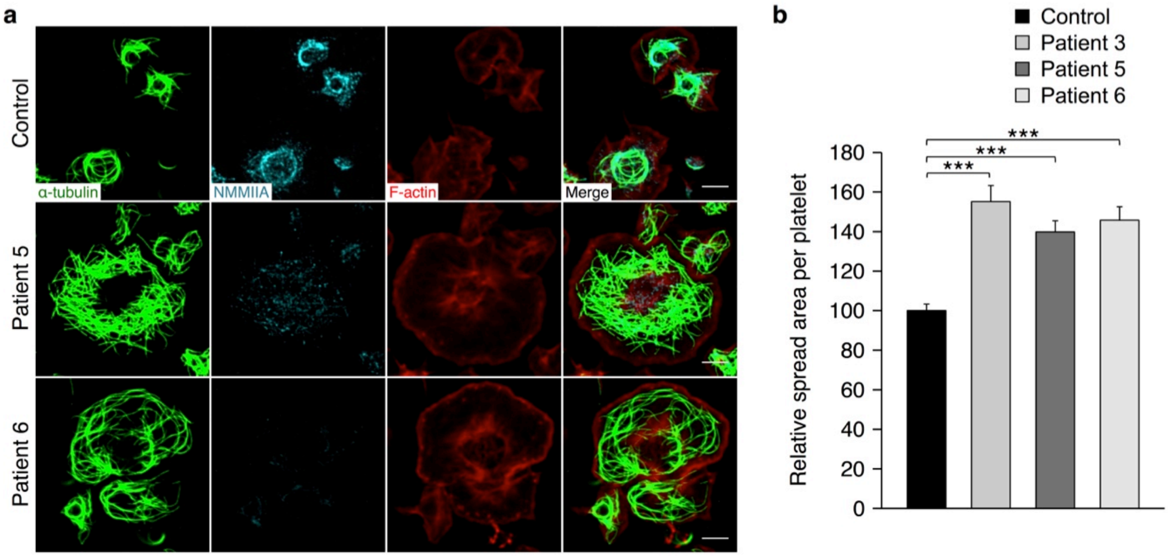

Supplementary Figure 23. Accelerated degradation of NMMIIA in spread platelets from patients with the *TRPM7* p.C721G variant. (**a**) Spread platelets (15 min, 100 μg mL^{-1} fibrinogen) from healthy controls and patients 5 and 6 were fixed and stained for α-tubulin (green), F-actin (red) and NMMIIA (cyan). Scale bars, 3 μm. (**b**) The relative spread surface area of platelets was determined using F-actin staining as a measure. Values are mean ± s.d. (n = 3 controls; at least 240 platelets). Unpaired Student's t-test; ****P*<0.001.

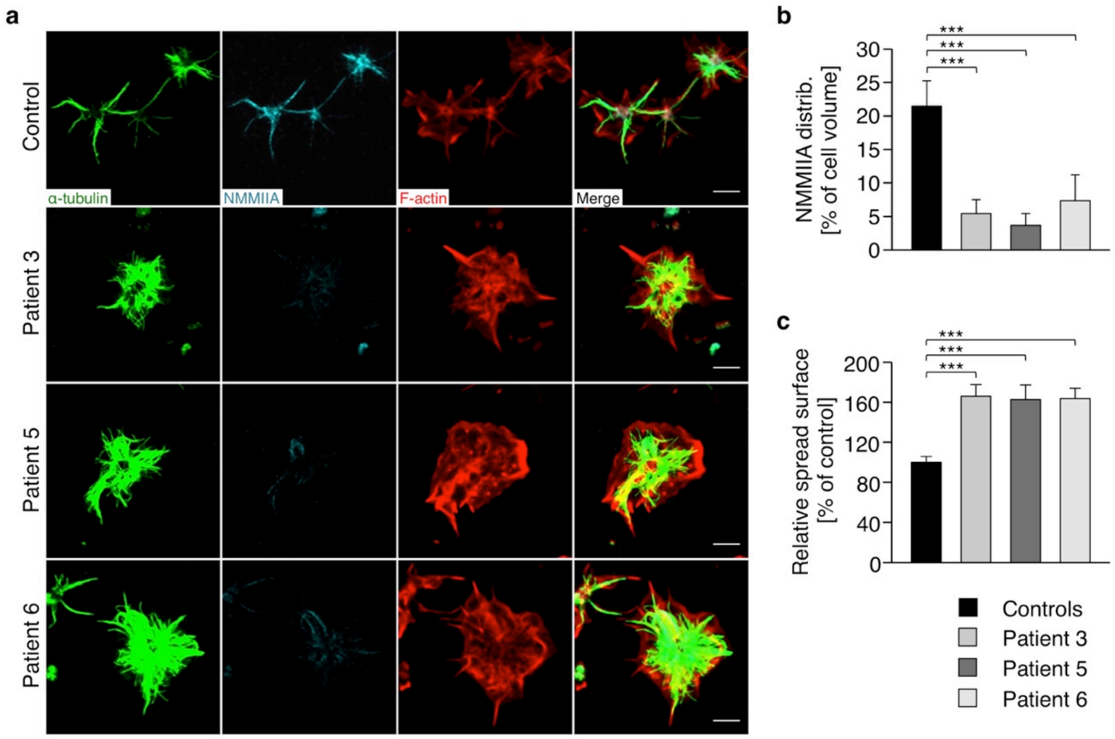

Supplementary Figure 24. Accelerated degradation of NMMIIA in spread platelets from patients with the *TRPM7* p.C721G variant. (**a**) Spread platelets (15 min, 10 μg mL^{-1} collagen-related peptide) of healthy controls, patient 3, 5 and 6 were fixed and stained for α-tubulin (green), F-actin (red) and NMMIIA (cyan). Scale bars, 3 μm. (**b**) Quantification of the relative NMMIIA content on confocal microscopy images of spread platelets. Values are mean ± s.d. (n = 3 controls; 100 platelets). (**c**) The relative spread surface area of platelets was determined using F-actin staining as a measure. Values are mean ± s.d. (n = 3 controls; at least 240 platelets). Unpaired Student's t-test; ***P<0.001.

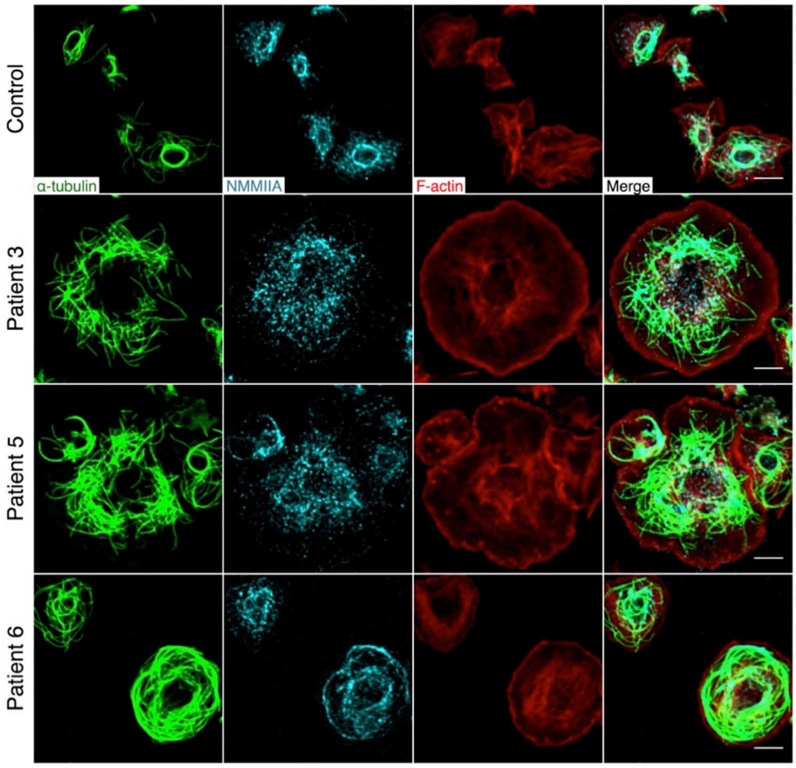

Supplementary Figure 25. Blebbistatin pretreatment prevented the degradation of NMMIIA in platelets from patients with the *TRPM7* p.C721G variant. Blebbistatin-pretreated (25 μM) spread platelets (15 min, 100 μg mL^{-1} fibrinogen) of healthy controls (n = 3), patient 3, 5 and 6 were fixed and stained for α-tubulin (green), F-actin (red) and NMMIIA (cyan). Scale bars, 3 μm.

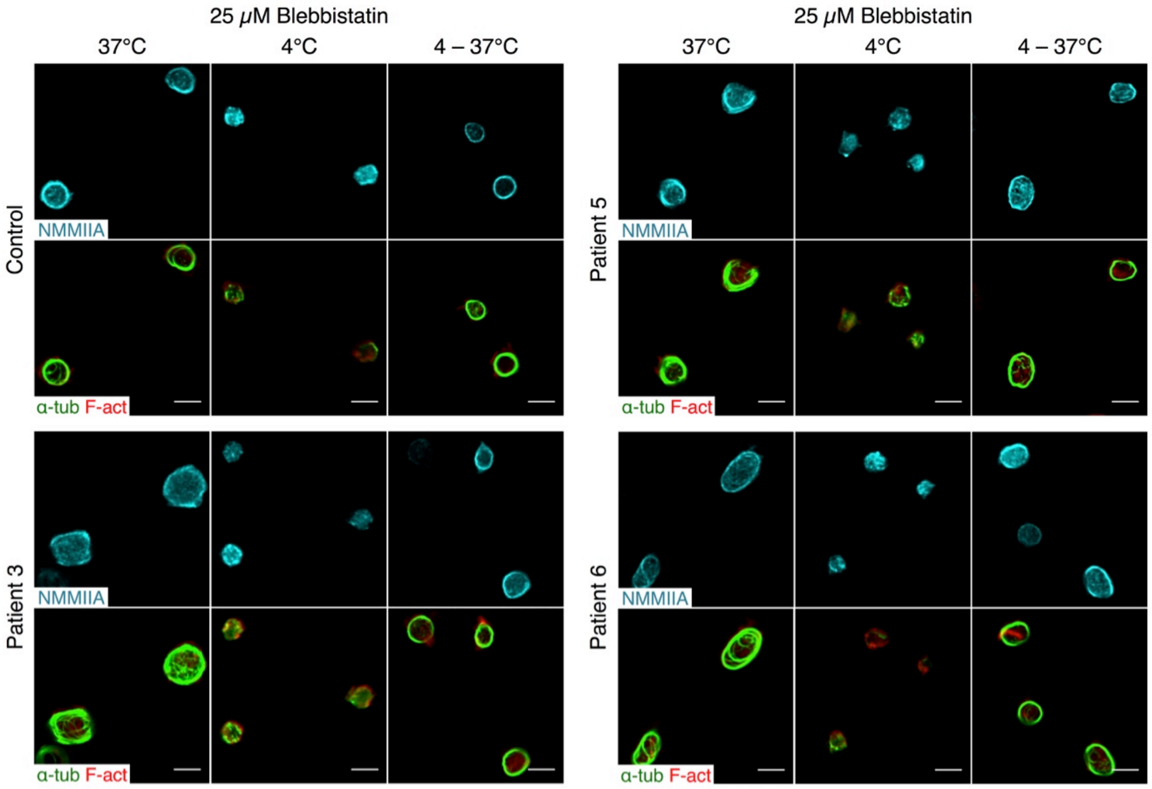

Supplementary Figure 26. Blebbistatin pretreatment restores the cytoskeletal architecture of platelets from patients with the *TRPM7* p.C721G variant upon rewarming. Resting platelets of healthy controls (n = 3), patient 3, 5 and 6 were subjected to cold challenge, fixed and stained for α-tubulin (green), F-actin (red) and NMMIIA (cyan). Scale bars, 3 μm.

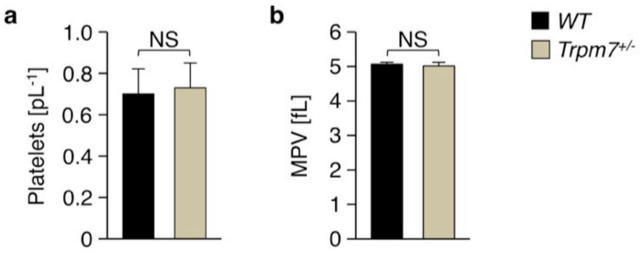

Supplementary Figure 27. Heterozygous *Trpm7* deficiency does not cause macrothrombocytopenia. (**a, b**) Peripheral platelet counts (**a**) and platelet volume (**b**) were quantified with an automated cell analyser. Values are mean ± s.d. (n = 7). Unpaired Student's t-test; NS, non-significant.

Fig. 3h

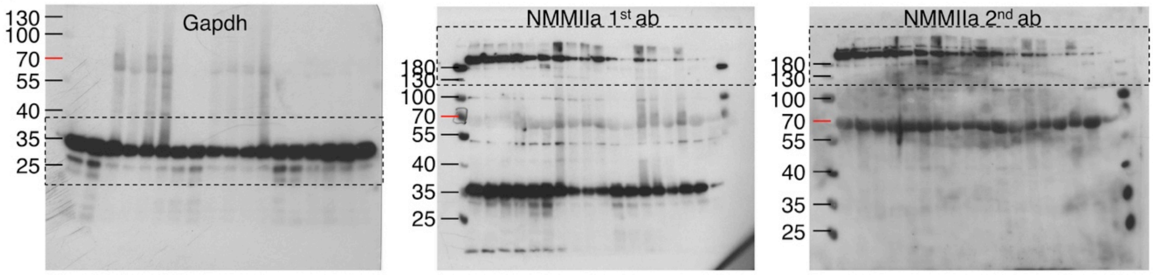

Fig. 4d, e

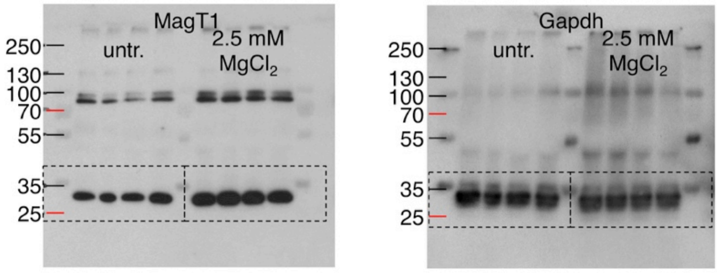

Fig. 5e

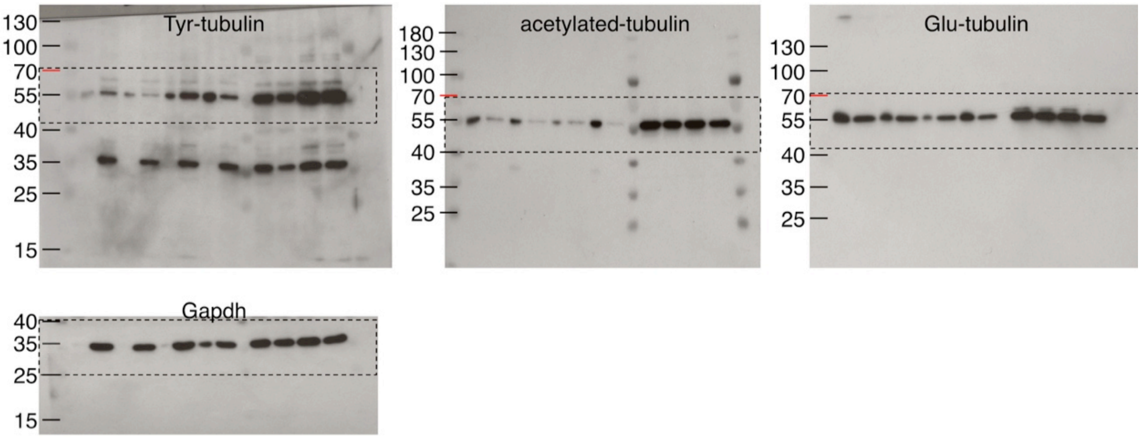

Supplementary Figure 28. Immunoblot images of selected bands displayed in main figures. Boxed areas were cropped for designated figures.

Supplementary Fig. 6c

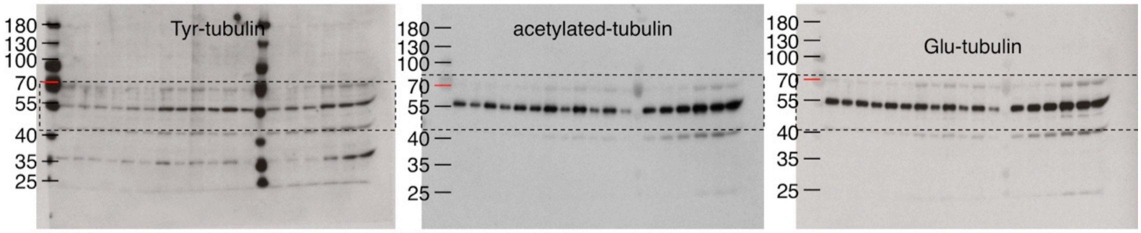

Supplementary Fig. 11c

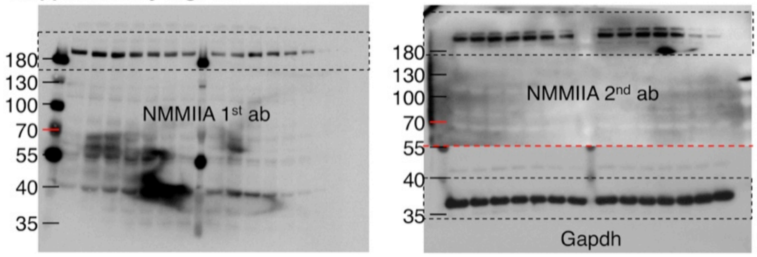

Supplementary Fig. 11g

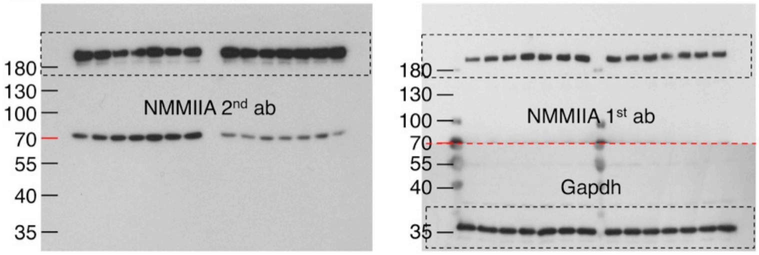

Supplementary Fig. 12a

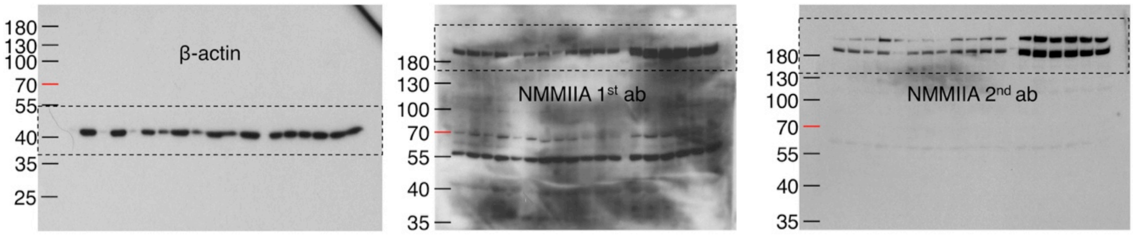

Supplementary Fig. 12b

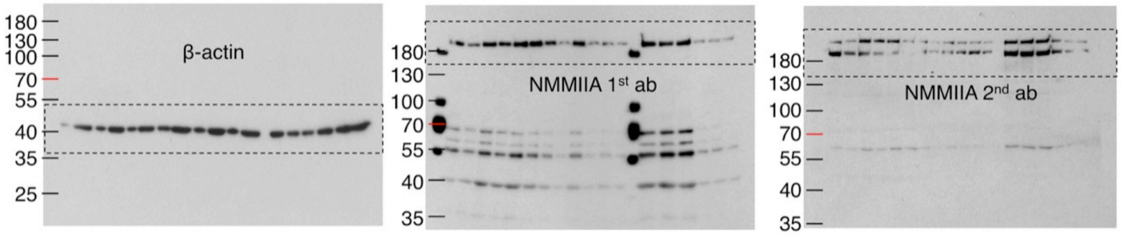

Supplementary Fig. 14c

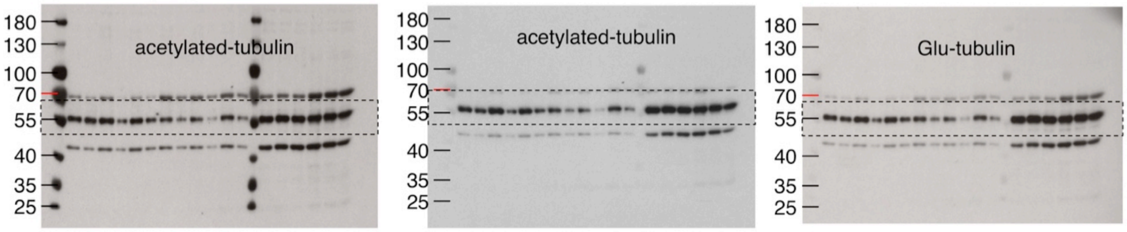

Supplementary Figure 29. Immunoblot images of selected bands displayed in supplementary figures. Boxed areas were cropped for designated figures.

Supplementary Table 1. BRIDGE-BPD study database entries and human phenotype ontology (HPO) coded clinical and laboratory characteristics of the three index cases with variants in *TRPM7*. Patient UCN 0025 presented with normal red blood cell numbers with a decreased mean globular volume. Due to her origin (Guadeloupe) this patient is suspected to suffer from beta-thalassemia.

**Unique case number (UCN)**	0012 (patient 5)	0025	0110
**Platelet count** (x10^9 L^{-1}; N: 150-400)	94	50	409
**Mean platelet volume** (fL; N: 7.0-10.0)	11.5	10.5	Not applicable
**HPO – Hematology terms**	Increased mean platelet volume, thrombocytopenia, normal platelet aggregation, no bleeding diathesis	Thrombocytopenia, qualitative abnormal hemoglobin, normal platelet aggregation, no bleeding diathesis	Impaired ADP-/epinephrine-induced platelet aggregation, abnormal platelet granules, subcutaneous hemorrhage, epistaxis, menorrhagia, post-partum hemorrhage, prolonged bleeding after dental extraction
**Variant coordinate**	50903409	50899402	50884374
**Reference allele**	T	C	G
**Alternate allele**	G	T	A
**Effect on amino acid sequence**	p.C721G (c.2161T>G)	p.R902C (c.2704C>T)	p.G1353D (c.4058G>A)
**Combined annotation dependent depletion (CADD) score**	24.80	20.40	15.68

Supplementary Table 2. Demographic information and blood parameters of the patient cohort. Blood parameters of patients 3, 5 and 6 were determined in ACD-A anticoagulated whole blood with the help of with an automated cell analyser (Sysmex).

**Pedigree**	**p.C721G**		
**Patient**	**3**	**5**	**6**
**Gender**	F	F	M
**Year of birth**	1964	1965	1985
**Platelet count** ($x10^9$ L^{-1}; N: 150-400)	100	80	123
**Mean platelet volume** (fL; N: 7.0-10.0)	12.3	11.2	11.1
**Plateletcrit** (%; N: 0.19-0.40)	0.12	0.09	0.14
**Red blood cell count** ($x10^{12}$ L^{-1}; N: 4.0-6.2)	5.15	5.25	5.41
**Hemoglobin** (g dL^{-1}; N: 12-18)	15.7	14.6	15.4
**Hematocrit** (%; N: 45.0-55.0)	44.1	43.1	46.1
**White blood cell count** ($x10^9$ L^{-1}; N: 4.0-10.0)	9.52	6.83	5.80
**Neutrophil count** ($x10^9$ L^{-1}; N: 1.5-7.0)	5.94	4.62	3.21
**Lymphocyte count** ($x10^9$ L^{-1}; N: 1.5-4.0)	3.01	1.50	1.72
**Monocyte count** ($x10^9$ L^{-1}; N: 0.1-1.0)	0.36	0.45	0.44
**Eosinophil count** ($x10^9$ L^{-1}; N: 0.1-0.7)	0.14	0.20	0.33
**Basophil count** ($x10^9$ L^{-1}; N: < 0.1)	0.07	0.06	0.10

Supplementary Table 3. Summary of primers used for Mg^{2+} channel and transporter expression profiling in murine platelets.

Genes	Primer sequence	Fragment size [bp]
MagT1	5´-tcggaccgtgctggaagaaa-3´<br>5´-gagctttaacaagacgacgg-3´	255
Tusc3	5´-tactggtagctttcccttcc-3´<br>5´-attcttcgttagcctgcctg-3`	263
Acdp1	5´-tgttcgtcaaagacttggcc-3´<br>5´-ggatctccgacttgatgatc-3´	261
Acdp2	5´-aagacttggccttcgtggat-3´<br>5´-acaggtctgtctcatccaag-3´	270
Acdp3	5´-ataccaaactggacgctgtc-3´<br>5´-cagacaccttgaataaggag-3´	267
Acdp4	5´-ctacactcgcattcctgtgt-3´<br>5´-gatgacgtcctccagagtga-3´	289
Nipa1	5´-tagtgaacgggtccacgttc-3´<br>5´-ttagcagacagcccaacttg-3´	267
Nipa2	5´-gaactactctgccgtggtta-3´<br>5´-tcatagccaatcccagacca-3´	262
Nipa3	5´-caatctgtatgtgggcttgg-3´<br>5´-ttatgagaacgctcagagcc-3´	233
Nipa4	5´-accttgatcacctggcaaga-3´<br>5´-tcgcaggtgcaaatgcatag-3´	259
Slc41A1	5´-ctccttttccattggactgc-3´<br>5´-atcatccgccagagctcctt-3´	246
Slc41A2	5´-catggctctgcagatattgg-3´<br>5´-gtatgatggctgccacagct-3´	311
Slc41A3	5´-gagacgtccctgatcattgg-3´<br>5´-catcgatttgccccagtgtt-3´	226
Trpm6	5´-tgtgggcggtgctcatgaag-3´<br>5´-caagccattcgtgcacgctg-3´	450
Trpm7	5´-gagcccaacagatgcttatgg-3´<br>5´-ggcccgccttcaaatatcaaag-3´	550
Actb	5´-gtgggccgctctaggcaccaa-3´<br>5´-ctctttgatgtcacgcacgatttc-3´	500

SUPPLEMENTARY NOTES:

Members of the NIHR-BioResource Rare Diseases consortium:

Name	Institution
**Principal Investigators BRIDGE Consortium Projects**	
Timothy Aitman	Imperial College/University of Edinburgh
David Bennett	University of Oxford/Oxford University Hospitals
Mark Caulfield	Queen Mary University of London
Daniel Gale	University College London
Ania Koziell	Guy's and St Thomas' NHS Foundation Trust
Taco W Kuijpers	Emma Children's Hospital AMC, Amsterdam
Eamonn Maher	University of Cambridge/Cambridge University Hospitals
Hugh S Markus	University of Cambridge/Cambridge University Hospitals
Nicholas Morrell	University of Cambridge/Cambridge University Hospitals
Willem H Ouwehand	University of Cambridge/ NHS Blood and Transplant/ Wellcome Trust Sanger Institute
David Perry	Cambridge University Hospitals
F Lucy Raymond	University of Cambridge/Cambridge University Hospitals
Irene Roberts	University of Oxford/Oxford University Hospitals NHS FT
Kenneth Smith	University of Cambridge/Cambridge University Hospitals
Adrian Thrasher	Great Ormond Street Hospital
Hugh Watkins	University of Oxford/Oxford University Hospitals NHS FT
Catherine Williamson	King's College London
Geoffrey Woods	University of Cambridge/Cambridge University Hospitals
**NIIHR BioResource - Rare Diseases - Management Team**	
Sofie Ashford	University of Cambridge
John R Bradley	Cambridge University Hospitals
Debra Fletcher	University of Cambridge
Tracey Hammerton	University of Cambridge
Roger James	University of Cambridge
Nathalie Kingston	University of Cambridge
Christopher J Penkett	University of Cambridge
Kathleen Stirrups	University of Cambridge
Marijke Veltman	University of Cambridge
Tim Young	University of Cambridge
**Enrolment and Ethics**	
Matthew Brown	University of Cambridge
John Davis	University of Cambridge
Eleanor Dewhurst	University of Cambridge
Amy Frary	University of Cambridge
Marie Erwood	University of Cambridge
Jennifer Martin	University of Cambridge
Sofia Papadia	University of Cambridge
Karola Rehnstrom	University of Cambridge
**BRIDGE-BPD Consortium**	
David Allsup	Department of Haematology, Castle Hill Hospital, Hull and East Yorkshire NHS Foundation Trust
Steve Austin	Department of Haematology, Guys and St Thomas' NHS Foundation Trust
Tamam Bakchoul	Institut für Immunologie und Transfusionsmedizin, Ernst-Moritz-Arndt-University of Greifswald, Greifswald

Tadbir K Bariana	The Katharine Dormandy Haemophilia Centre and Thrombosis Unit, Royal Free London NHS Foundation Trust/University College London
Paula Bolton-Maggs	NHS Blood and Transplant, Manchester
Elizabeth Chalmers	Royal Hospital for Children, NHS Greater Glasgow and Clyde
Peter Collins	Arthur Bloom Haemophilia Centre, University Hospital of Wales Heath Park, Cardiff, Wales
Wendy N Erber	Pathology and Laboratory Medicine, University of Western Australia, Crawley, Western Australia
Tamara Everington	Salisbury Hospital, Salisbury NHS Fondation Trust
Kathleen Freson	Department of Cardiovascular Sciences, Center for Molecular and Vascular Biology, University of Leuven
Bruce Furie	Beth Israel Deaconess Medical Centre, Harvard Medical School, Boston
Michael Gattens	Cambridge University Hospitals NHS Foundation Trust
Keith Gomez	The Katharine Dormandy Haemophilia Centre and Thrombosis Unit, Royal Free London NHS Foundation Trust/University College London
Daniel Greene	Department of Haematology, University of Cambridge/MRC-BSU
Andreas Greinacher	Institute for Immunology and Transfusion Medicine, Ernst-Moritz-Arndt-University of Greifswald, Greifswald
Daniel Hart	The Royal London Hospital, Barts Health NHS Foundation Trust
Johan WM Heemskerk	Maastricht University, Maastricht
Yvonne Henskens	Maastricht University Medical Centre, Maastricht
Rashid Kazmi	Southampton General Hospital, University Hospital Southampton NHS FT
David Keeling	Oxford Haemophilia and Thrombosis Centre, Oxford University Hospitals NHS Trust, The Churchill Hospital, Oxford
Anne M Kelly	Cambridge University Hospitals NHS Foundation Trust
Claire Lentaigne	Imperial College Healthcare NHS Trust/Imperial College London
Ri Liesner	Department of Haematology, Great Ormond Street Hospital for Children NHS Trust, London
Sarah Mangles	Haemophilia, Haemostasis and Thrombosis Centre, Hampshire Hospitals NHS Foundation Trust, Aldermaston Road, Basingstoke
Mary Mathias	Department of Haematology, Great Ormond Street Hospital for Children NHS Trust, London
Carolyn M Millar	Imperial College Healthcare NHS Trust/Imperial College London
Andrew Mumford	University of Bristol/University Hospitals Bristol NHS Foundation Trust
Jeanette Payne	Department of Haematology, Sheffield Children's Hospital NHS Foundation Trust
John Pasi	Barts and The London School of Medicine and Dentistry, Haemophilia Centre, The Royal London Hospital, London
David J Perry	Cambridge University Hospitals NHS Foundation Trust
Kathelijne Peerlinck	Department of Cardiovascular Sciences, Center for Molecular and Vascular Biology, University of Leuven
Michael Richards	Leeds Teaching Hospitals NHS Foundation Trust, Leeds
Matthew Rondina	Madsen Health Center, Salt Lake City
Catherine Roughley	Haemophilia Centre, Kent & Canterbury Hospital, East Kent Hospitals University Foundation Trust
Sol Schulman	Beth Israel Deaconess Medical Centre, Harvard Medical School, Boston
Marie Scully	University College London Hospital
Suthesh Sivapalaratnam	The Royal London Hospital, Barts Health NHS Foundation Trust
R Campbell Tait	Glasgow Royal Infirmary, NHS Greater Glasgow and Clyde
Kate Talks	Haematology Department, Royal Victoria Infirmary, Newcastle upon Tyne
Jecko Thachil	Haematology Department, Manchester Royal Infirmary, Manchester
Cheng-Hock Toh	The Roald Dahl Haemophilia Centre, Royal Liverpool Hospital, Liverpool
Chris Van Geet	Department of Cardiovascular Sciences, Center for Molecular and Vascular Biology, University of Leuven
Minka De Vries	Maastricht University Medical Centre, Maastricht
Timothy Q Warner	Barts Health NHS Foundation Trust
Sarah Westbury	University of Bristol/University Hospitals Bristol NHS Foundation Trust

Cambridge Translational GenOmics Laboratory

James C Fox	University of Cambridge
Abigail Furnell	University of Cambridge
Rutendo Mapeta	University of Cambridge
Ilenia Simeoni	University of Cambridge
Simon Staines	University of Cambridge
Jonathan Stephens	University of Cambridge
Deborah Whitehorn	University of Cambridge
**Clinical Bioinformatics**	
Antony Attwood	University of Cambridge
Louise Daugherty	University of Cambridge
Sri VV Deevi	University of Cambridge
Csaba Halmagyi	University of Cambridge
Fengyuan Hu	University of Cambridge
Vera Matser	University of Cambridge
Stuart Meacham	University of Cambridge
Karyn Megy	University of Cambridge
Catherine Titterton	University of Cambridge
Salih Tuna	University of Cambridge
Ping Yu	University of Cambridge
Julie von Ziegenweldt	University of Cambridge
**Genetic Epidemiology**	
William Astle	University of Cambridge
Marta Bleda	University of Cambridge
Keren Carss	University of Cambridge
Stefan Graf	University of Cambridge
Matthias Haimel	University of Cambridge
Hana Lango-Allen	University of Cambridge
**MRC Biostatistics Unit**	
Sylvia Richardson	University of Cambridge
**High Performance Computing Service**	
Paul Calleja	University of Cambridge
Stuart Rankin	University of Cambridge
Wojciech Turek	University of Cambridge
**Administrative Support**	
Christine Bryson	University of Cambridge
Sandie Campin	University of Cambridge
Coleen McJannet	University of Cambridge
Sophie Stock	University of Cambridge
**SPEED**	
Manju Kurian	Institute of Child Health
Detelina Grozeva	University of Cambridge (CIMR Medical Genetics)
Andrew Webster	Moorfields Hospital
Anthony Moore	Moorfields Hospital
Alasdair Parker	University of Cambridge/Cambridge University Hospitals
Dragana Josifova	Guy's and St Thomas' NHS Foundation Trust
Julia Rankin	Royal Devon & Exeter NHSFT
Evangeline Wassmer	Birmingham Children's Hospital
Natalie Canham	North West Thames NHS
Emma Wakeling	North West Thames NHS
Maria Bitner-Glindzicz	Great Ormond Street Hospital
Richard Scott	Great Ormond Street Hospital

**PAH**	
Andrew Peacock	Golden Jubilee National Hospital
David Kiely	Sheffield CRF, Royal Hallamshire
Gerry Coghlan	Royal Free
Joanna Pepke-Zaba	Papworth
Martin Wilkins	Imperial and Hammersmith
Paul Corris	Newcastle Freeman
Shahin Moledina	GOSH
John Wort	Royal Brompton
Jay Suntharalingam	Bath
Harm Boggard	VU University Medical Center, Amsterdam
John Wharton	Imperial and Hammersmith
Rob Mackenzie	Bath
Mark Toshner	Papworth
Allan Lawrie	Sheffield CRF, Royal Hallamshire
Richard Trembath	Kings College, London
Simon Gibbs	Imperial and Hammersmith
Anton Vonk Noordegraaf	VU University Medical Center, Amsterdam
Colin Church	Golden Jubilee National Hospital
**PID**	
Chiara Bacchelli	UCL
Emily Staples	Addenbrooke's Hospital/Cambridge University
Hans Stauss	UCL/Royal Free Hospital
Helen Baxendale	Papworth Hospital
Hilary Longhurst	Barts Health NHS Trust
James Thaventhiran	University of Cambridge
Jesmeen Maimaris	Insitute of Child Health, UCL
Kimberley Gilmour	GOSH
Rainer Doffinger	University of Cambridge
Richard Antrobus	Birmingham Queen Elizabeth Hospital
Siobhan Burns	Royal Free Hospital
Suranjith Seneviratne	Royal Free Hospital
